# Advances in Phage Therapy: Targeting the *Burkholderia cepacia* Complex

**DOI:** 10.3390/v13071331

**Published:** 2021-07-09

**Authors:** Philip Lauman, Jonathan J. Dennis

**Affiliations:** Department of Biological Sciences, University of Alberta, Edmonton, AB T6G 2E9, Canada; lauman@ualberta.ca

**Keywords:** *Burkholderia cepacia* complex (Bcc), bacteria, pathogenesis, antibiotic resistance, bacteriophages, phages, phage therapy, phage therapy treatment strategies, Bcc phage therapy

## Abstract

The increasing prevalence and worldwide distribution of multidrug-resistant bacterial pathogens is an imminent danger to public health and threatens virtually all aspects of modern medicine. Particularly concerning, yet insufficiently addressed, are the members of the *Burkholderia cepacia* complex (Bcc), a group of at least twenty opportunistic, hospital-transmitted, and notoriously drug-resistant species, which infect and cause morbidity in patients who are immunocompromised and those afflicted with chronic illnesses, including cystic fibrosis (CF) and chronic granulomatous disease (CGD). One potential solution to the antimicrobial resistance crisis is phage therapy—the use of phages for the treatment of bacterial infections. Although phage therapy has a long and somewhat checkered history, an impressive volume of modern research has been amassed in the past decades to show that when applied through specific, scientifically supported treatment strategies, phage therapy is highly efficacious and is a promising avenue against drug-resistant and difficult-to-treat pathogens, such as the Bcc. In this review, we discuss the clinical significance of the Bcc, the advantages of phage therapy, and the theoretical and clinical advancements made in phage therapy in general over the past decades, and apply these concepts specifically to the nascent, but growing and rapidly developing, field of Bcc phage therapy.

## 1. Introduction

The increasing prevalence and global spread of multidrug-resistant bacterial pathogens is an imminent danger to public health and threatens virtually all aspects of modern medicine. Antibiotic resistance is a major cause of morbidity and mortality in developed and developing countries alike, with 35,000 premature deaths due to drug resistance occurring per annum in the United States alone, and treatment options are in many cases becoming limited [1]. A 2019 report by the Canadian Council of the Academies revealed that 26% of all bacterial infections in 2018 were resistant to the drugs used to treat them, but estimated that this percentage will rise to 40% by the year 2050, when it is predicted that drug resistance will cause 10 million premature fatalities globally per annum and cost an approximated USD 100 trillion in damages [2,3]. Discovery of novel antibiotics with new cellular targets has failed to keep pace with the evolution of resistance mechanisms in bacteria, partially because antibacterials are less profitable than other pharmaceutical products, and resistance is now developing to antibiotics of last resort, such as colistin [4,5]. Even if novel antibiotics are developed, resistance to them will inevitably arise, due to the genetic nature of bacterial populations, unless these antibiotics are used strategically and in combination with other medications. Clearly, novel and distinct modes of treatment are desperately required.

One promising alternative to antibiotic therapy is phage therapy—the medicinal application of bacterial viruses called bacteriophages (phages). Bacteriophages are environmentally abundant, outnumbering bacteria by approximately ten-fold, and are estimated to destroy approximately 50% of the global bacterial population every forty-eight hours, meaning that phage therapy is simply the application of a naturally occurring antibacterial agent to a human problem [6,7]. In addition to their powerful antibacterial properties, phages have a number of major advantages over chemical antibiotics, including a lack of side effects, exquisite target specificity, virtually limitless environmental abundance, and advantageous pharmacokinetics. Importantly, the mode of action of bacteriophages is entirely distinct from that of antibiotics—meaning that bacteria that are resistant to antibiotics remain susceptible to bacteriophages [8]. Discovered in the early 20th century, phages were immediately employed to treat diseases by their discoverer, Felix d’Herelle, and although they were largely replaced in Western nations in the 1940s, by broad spectrum antibiotics, phage therapy remained a viable form of treatment in many Eastern Bloc countries, and their use in several of these nations is ongoing [9].With decreasing antibiotic drug discovery and increasing antibiotic resistance, research into phage therapy is now in the midst of a renaissance that began in the mid-1980s, with phages now being tested, using various therapeutic strategies, against a growing list of increasingly dangerous and drug-resistant bacterial pathogens. 

Among the most clinically challenging of these pathogens are the members of the *Burkholderia cepacia* complex (Bcc), a group of over twenty species that possess high levels of innate antibiotic resistance and that cause severe disease in patients who are immunocompromised or afflicted with certain diseases [10]. The success rates of antibiotic therapy against these pathogens are generally poor and the affected patients are often left with few options, meaning that the development of a novel approach to combating these pathogens—such as phage therapy—is required. In this report, we discuss the notorious Bcc, review the theoretical and clinical advancements made in phage therapy over the past decades, and apply these concepts to the nascent, but growing and rapidly developing, field of Bcc phage therapy.

## 2. Bacteria of the *Burkholderia cepacia* Complex

**Figure 1:**
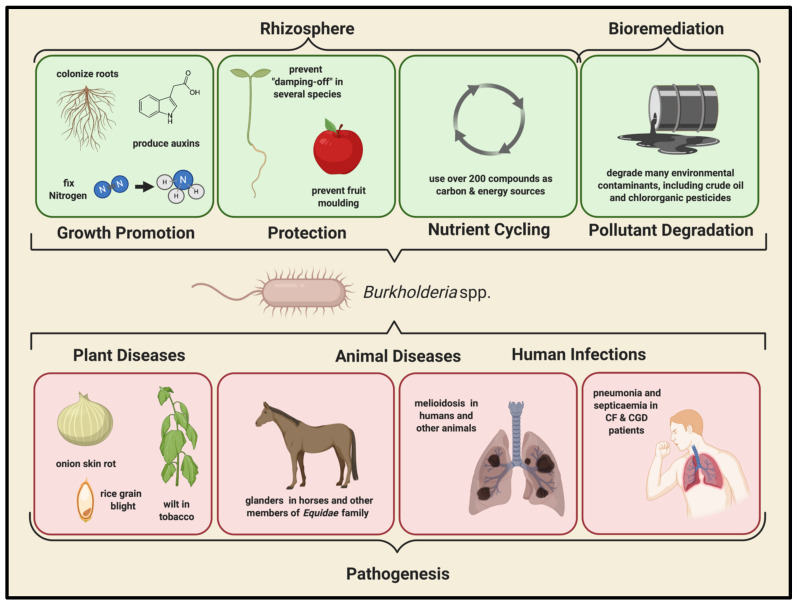
Environmental and pathogenic roles of *Burkholderia* species. Members of the genus *Burkholderia* have a wide range of roles in both environmental and clinical settings. Although many species directly promote plant growth, secrete antibacterial and antifungal agents to protect plants from infection from other organisms, and contribute heavily to soil nutrient flux, they are also known to cause disease in a number of crops including onions, tobacco, rice, as well as several species of flowering plants. Due to their impressive versatility with respect to carbon and energy sources, *Burkholderia* species have been investigated for use in bioremediation, and are known to be capable of breaking down several difficult-to-degrade contaminants. Unfortunately, several species within this genus are known to cause severe diseases in both human and non-human animals—including *B. mallei*—which causes glanders in horses and other animals; *B. pseudomallei*, the causative agent of melioidosis in humans and other animals; and members of the Bcc, which cause persistent, difficult-to-treat and often fatal lung infections in susceptible human populations.

The bacterium *Pseudomonas cepacia* (the founding and eponymous member of what is now the *B. cepacia* complex, Bcc) was initially discovered as the causative agent of onion bulb sour skin rot in 1950 by Walter Burkholder, who noted that rotted onion bulbs infected by *P. cepacia* had an odoriferous, yellow appearance that was distinct from that seen in fungal and *Pseudomonas allicola* infections. Burkholder described the bacterium as an obligately aerobic, flagellated, Gram-negative rod that is capable of using a diverse array of nutrients as carbon, nitrogen and energy sources for growth and metabolism [11]. This feature is shared by many other members of the Bcc and contributes to the versatility of these organisms—allowing them to adapt and flourish in a variety of environments. Indeed, the Bcc members are ubiquitous in nature and have been isolated from a wide range of niches, including human sputum and tissue samples, hospital surfaces, medical devices, water, soil, and the tissues and rhizospheres of various plants [10,12,13,14,15,16,17] (Figure 1).

As novel species that are closely related to *P. cepacia* were progressively isolated and characterized in the late 20th century, a novel genus—*Burkholderia*—was ultimately proposed for these organisms, by Yabuuchi et al. in 1992. This genus originally contained seven species—*B. cepacia*, *Burkholderia mallei*, *Burkholderia pseudomallei*, *Burkholderia caryophylli*, *Burkholderia gladioli*, *Burkholderia pickettii*, and *Burkholderia solanacearum*—which were moved from rRNA group II of the *Pseudomonas* genus based on 16s rRNA sequences, DNA–DNA homology, cell-membrane lipid and fatty acid composition, and various phenotypic characteristics, and additional *Pseudomonas* species were later reclassified as *Burkholderia* species based on the results of DNA–rRNA hybridization studies [18,19]. Phenotypically similar, but genotypically distinct, strains of *B. cepacia* were later divided into five distinct genomovars, which, together, composed what is now called the *B. cepacia* complex (Bcc), and four additional genomovars were subsequently added [20,21,22,23]. Over the next two decades, these genomovars were renamed as distinct species—*B. cepacia*, *Burkholderia multivorans*, *Burkholderia cenocepacia*, *Burkholderia stabilis*, *Burkholderia vietnamiensis*, *Burkholderia dolosa*, *Burkholderia ambifaria*, *Burkholderia anthina*, and *Burkholderia pyrrocinia*—and additional related species, primarily isolated from environmental sources, were progressively added to the growing Bcc—bringing the total number of genotypically distinct Bcc species to at least twenty-four [10,24,25,26,27,28,29].

### 2.1. Environmental Importance of the Bcc

The bacteria of the Bcc are found ubiquitously in the natural environment, and have been isolated from soil, water sources, and the tissues and rhizospheres of many plants, including a number of different crops [10]. In these environments, many *Burkholderia* and Bcc species have been identified as plant pathogens, including several Bcc members that cause skin rot in onions, *B. caryophylli*, *B. gladioli* and *Burkholderia glumae*—which cause bacterial wilt in carnations, gladioli, iris flowers, and tobacco [30,31]—and *B. gladioli*, *B. glumae*, and *Burkholderia plantarii*—which cause blight and rot of rice grains and seedlings [32,33,34]. Conversely, many Bcc members have symbiotic relationships with certain plants, and exhibit a number of beneficial behaviors that promote plant growth, including nitrogen fixation, auxin production, nutrient mobilization, and root colonization, which facilitates plant nutrient absorption [35,36,37,38]. Several Bcc members are also able to suppress soil-borne diseases via antagonistic effects against certain soil-borne pathogens [39,40], and can additionally inhibit fungal growths through the production of antifungal compounds, such as pyrrolnitrin, which has been demonstrated to inhibit mold growth on fruit [41]. Finally, several Bcc species are able to prevent damping-off disease in seedlings, caused by *Rhizoctonia solani* and various *Pythium* species, which affects a wide variety of crop plants worldwide [42]. 

In addition to their interactions with plants, the Bcc species play a major role in environmental nutrient cycling, due to their metabolic diversity. Indeed, these bacteria are able to utilize over 200 distinct organic compounds as carbon and energy sources, including a large variety of alcohols, carboxylic acids, amines, amino acids, and even aromatic compounds, and have been shown to degrade various environmental pollutants and groundwater contaminants, including, remarkably, crude oil [12,13,16,43,44,45,46,47,48,49,50,51]. Consequently, many Bcc species were used commercially as biocontrol and bioremediation agents in the United States, but a moratorium on the agricultural use of these biopesticidal species was implemented in 1999, due to their highly problematic role as multidrug-resistant, opportunistic pathogens that are implicated in human disease [52,53].

### 2.2. Clinical Importance of the Bcc

Although the Bcc members do not generally infect healthy individuals, these bacteria are strongly associated with respiratory tract infections in immunocompromised individuals, such as those suffering from chronic granulomatous disease (CGD), and patients afflicted with cystic fibrosis (CF; see Section 2.3). Indeed, Bcc bacteria have emerged as important and highly opportunistic pathogens in both CF and CGD patients over the past 40 years, and are associated with rapid community spread, unusually aggressive infections, and high mortality rates in both of these patient groups [54,55,56,57].

*B. cepacia* began to be isolated from the respiratory tracts of CF patients with an increasing frequency in the 1980s, possibly as a result of the improved identification methods, and these initial reports described the unusually virulent nature of many *B. cepacia* infections. Indeed, while the Bcc members account for a relatively small proportion of pulmonary infections in CF patients, the majority of such infections being caused by *Pseudomonas aeruginosa*, Bcc-afflicted patients have poorer lung function and higher mortality rates relative to patients infected by *P. aeruginosa* [54,58,59,60]. Similarly, although Bcc infections account for only a small number of respiratory infections in CGD patients, they are remarkably aggressive and have the highest fatality rates in these individuals [56,57]. In many cases, these high mortality rates are the result of a disease progression termed “*cepacia* syndrome”, which is in direct contrast to the progressions seen in chronic infections caused by other CF and CGD pathogens, and is characterized by high fever, rapid and severe progressive respiratory failure, decline in leukocyte and erythrocyte levels, necrosis, bacteremia, and sepsis, resulting in rapid death [61]. While the precise mechanisms leading to *cepacia* syndrome are likely multifactorial and remain incompletely understood, in vitro studies have demonstrated that ROS-deficient neutrophils from CGD patients are unable to destroy *B. cenocepacia* after phagocytic internalization, and are subsequently destroyed from within. This form of necrotic neutrophil death permits the leakage of toxic intracellular contents into tissues, thereby causing further tissue damage, perpetuation of the inflammatory response, and sepsis, which may at least partially explain *cepacia* syndrome and the high mortality rate of Bcc infections in CGD patients [57]. 

In addition to being highly virulent, at least five Bcc species—*B. cepacia*, *B. cenocepacia*, *B. multivorans*, *B. dolosa*, and *Burkholderia contaminans*—are capable of being disseminated via aerosol droplets, meaning that these pathogens can spread rapidly among susceptible patients in nosocomial settings, through direct and indirect contact with infected individuals [10,60]. Several strains of *B. cenocepacia* are known to have spread through social contact of this type, including electrophoretic type 12 (ET12), which has caused deadly epidemics among CF patients in Eastern Canada and the United Kingdom between 1986 and 1992, and the Philadelphia District of Columbia (PHDC) strain, which is associated with CF patients in the Eastern United States [62,63]. Furthermore, both of these strains have been isolated from CF patients in the UK, France, and Italy, suggesting that they are widely distributed [64]. Due to the high potential for these organisms to spread through susceptible populations, Bcc-positive patients are asked to follow stringent infection control practices and are isolated from other patients who are afflicted with CF in clinics—these are measures that have serious social and psychological ramifications that can potentially exacerbate patient illness [65,66,67]. Since the Bcc species are able to survive on various abiotic surfaces for several hours, Bcc infection may also be contracted through contact with contaminated hospital surfaces and objects, including nebulizers, nasal sprays, ultrasound gels, disinfectants, as well as hospital water [17,68]. Disturbingly, several Bcc members have recently been observed to possess an intrinsic resistance to benzalkonium chloride, a compound found in most commercial disinfectants and industrial sanitizers, which makes Bcc bacteria one of the most challenging microorganisms to eradicate, and increases the risk of contracting them in nosocomial settings [69]. Although there have been no reports to definitively prove patient acquisition of Bcc infections from the environment, Bcc bacteria are routinely isolated from environmental sources such as soil and water, implying that this route of transmission is possible [70].

Although most Bcc species are able to colonize the lungs of CF patients, infection by *B. multivorans* and *B. cenocepacia* is the most common, accounting for approximately 75% of all Bcc infections in patients afflicted with CF. *B. cenocepacia* has been recovered most frequently in Canadian and Western European CF patients, but there has been a shift in prevalence to *B. multivorans* in recent years, particularly in the United States. However, while *B. multivorans* accounts for a large number of infections, community spread of this species among CF patients is recorded less frequently than for *B. cenocepacia* [71,72]. CF patients infected by Bcc species are sometimes co-infected with other opportunistic pathogens, notably *P. aeruginosa* and less frequently *Stenotrophomonas maltophilia*, which may be predictive of poorer disease progression, since *B. cenocepacia*–*P. aeruginosa* co-infection has been shown to increase inflammation and biofilm mass in murine infection models [73,74]. Furthermore, while transient co-infections by different Bcc species or strains sometimes occur in CF patients, chronic Bcc infections in these patients are almost always due to a single strain [75]. Conversely, no single species or strain has been shown to dominate in Bcc infections of CGD patients, and epidemiological studies have found that recurrent pulmonary infections with distinct *Burkholderia* strains are common in these individuals. Interestingly, CGD patients are known to have been infected by Bcc species that are rarely seen in human infections, including *B. ambifaria*, *Burkholderia metallica* and, surprisingly, the rice pathogen *B. glumae* [10,56,76].

### 2.3. Bcc Infections

As opportunistic, obligate pathogens in humans, Bcc species very rarely infect healthy individuals, but are able to cause severe disease among immunocompromised populations, including young children, elderly people, pregnant women, and patients afflicted with cancer or other chronic illnesses. The vast majority of Bcc infections are hospital-acquired or healthcare-associated, and disproportionately affect males, especially in non-CF patient populations [77,78]. Representing the overwhelming majority of Bcc infection cases, the two largest at-risk populations are patients afflicted with cystic fibrosis (CF) or chronic granulomatous disease (CGD), among whom the rates of severe clinical deterioration and subsequent fatality are the highest [10,57]. 

#### 2.3.1. Cystic Fibrosis (CF)

CF is an autosomal recessive disorder caused by mutations in the gene encoding the cystic fibrosis transmembrane conductance regulator (CFTR), which primarily functions as a chloride channel, but additionally plays several regulatory roles, including, importantly, the inhibition of the epithelial Na^+^ channel (ENac)—which facilitates intracellular sodium transport from the extracellular space. Although several alternative models have been proposed, the prevailing mechanistic model of CF suggests that dysregulation of the CFTR causes increased epithelial intake of sodium, which is followed osmotically by water, leading to dehydration of the epithelial surface and the production of a thick, obstructive mucus [79,80,81]. Defects in the CFTR cause abnormalities in electrolyte transfer across the epithelial cells in general, leading to pathology in the lungs, pancreas, kidneys, liver, and gastrointestinal tract, but the majority of CF-related deaths are due to mucus buildup in the lungs, which inhibits mucociliary clearance and thus provides favorable conditions for colonization by certain bacterial species, such as the Bcc [10,79,82,83]. Furthermore, hypoxic cell death, due to mucus plugging of the airways, is thought to increase local inflammation, further exacerbating patient illness [81]. Although antibiotic therapy is able to reduce the severity of some bacterial infections, infections by the Bcc species are notoriously challenging to eliminate, due to the high levels of innate antimicrobial resistance. CF is the most frequently inherited lethal disease among Caucasians, affecting approximately 1 in 3000 individuals in the Caucasian populations of North American and European countries, and greatly decreases life expectancy (~40 years of age in developed nations) due to persistent and often untreatable infections [10,84,85,86]. 

#### 2.3.2. Chronic Granulomatous Disease (CGD)

CGD refers to a group of hereditary diseases caused by mutations in the genes that are involved in the production of reactive oxygen species (ROS), particularly the nicotinamide adenine dinucleotide phosphate (NADPH) encoding gene NOX2, which is essential for the production of the superoxide radical. ROS are critical for the intracellular destruction of endocytosed bacteria by neutrophils and macrophages, and the inability to produce these compounds results in increased susceptibility to certain pathogens, increased recruitment of phagocytes to infection sites, and prolonged, highly damaging inflammatory responses, at least partially caused by phagocyte necrosis [56,57,87,88]. No cures are available for CGD, but the severity of bacterial infections may be reduced through treatment with antibiotics and interferon-γ. Although CGD is relatively rare, affecting 1 in 200,000 to 250,000 individuals, patient life expectancy is significantly reduced (~45 years of age in developed countries) due to infections that are chronic and challenging to eradicate [88,89,90,91]. 

### 2.4. Antibiotic Resistance of the Bcc

**Figure 2:**
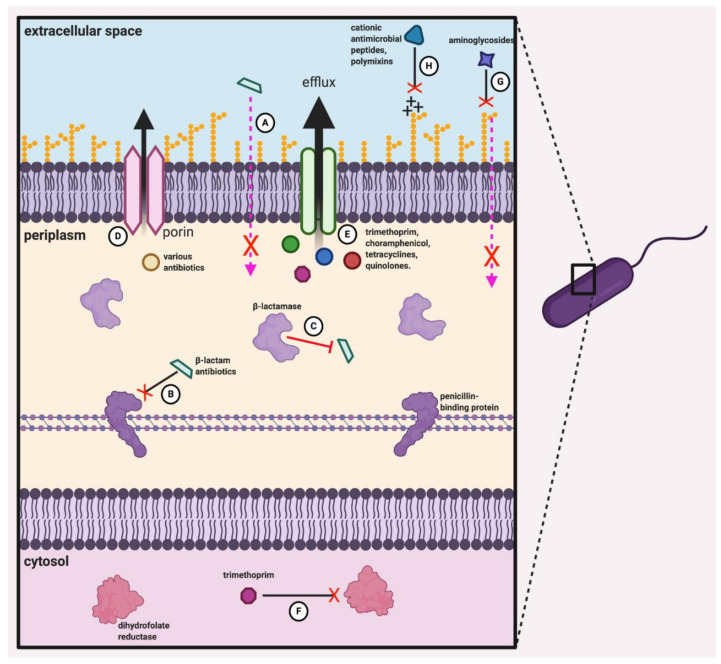
Antibiotic resistance mechanisms of the *Burkholderia cepacia* complex. Bcc members are resistant to a wide range of antibiotic compounds as a result of their large repertoire of both innate and acquired resistance mechanisms. Resistance to β-lactam antibiotics is achieved through a combination of reduced membrane permeability (**A**), mutated penicillin-binding proteins (**B**), and chromosomally encoded β-lactamases (**C**). Extrusion via porins (**D**) and efflux pumps (**E**) are responsible for resistance to a large number of compounds, including trimethoprim, chloramphenicol, tetracyclines and certain quinolones, while mutation of dihydrofolate reductase provides additional resistance to trimethoprim (**F**). At the outer membrane, LPS modifications prevent aminoglycoside binding and therefore block the trafficking of these compounds into the cell (**G**), while the reduced negative charge of the outer membrane reduces the binding of polymyxins and cationic antimicrobial peptides (**H**), thereby rendering Bcc species less vulnerable to these compounds.

*Burkholderia* species, including members of the Bcc, are notorious for their extremely high levels of innate resistance to a wide variety of antimicrobial compounds, which severely limits therapeutic options for the afflicted patients. The Bcc species are intrinsically resistant to β-lactams, aminoglycosides, cationic antimicrobial peptides, and polymyxins, and have multiple mechanisms of resistance to several other classes of antibiotics, including quinolones, tetracyclines, chloramphenicol, and trimethoprim [92,93,94,95] (Figure 2). Antibiotic resistance in the Bcc species can be attributed to a number of primary cellular resistance mechanisms, including altered drug targets and enzymatic modification of antimicrobials, which provides resistance to specific antibiotics or classes thereof, as well as low membrane permeability and drug extrusion via efflux pumps, which provide broad-spectrum protection against different classes of antibiotics [10,96]. Resistance to β-lactams is largely due to enzymatic modifications and altered drug targets, as most Bcc members possess chromosomally encoded β-lactamases and have altered penicillin-binding proteins [97,98]. Interestingly, far from being inhibited, some Bcc species are capable of growing on and using penicillin G as their sole carbon source [16,99]. An altered dihydrofolate reductase enzyme, which is the target of trimethoprim, confers resistance to this compound in a number of Bcc strains [100]. In *Burkholderia* species, the outer membrane has decreased permeability relative to many other bacteria, and the structure of the LPS is unique—a combination that provides resistance to several classes of antibiotics, including β-lactams, aminoglycosides, and cationic antimicrobial peptides [92,100]. Modifications in the LPS of the *Burkholderia* species prevents the binding of aminoglycosides, which require this binding for entry into the cell, and reduce the anionic charge of the cell surface, which inhibits the binding and subsequent bactericidal effects of cationic antimicrobial peptides [10,101,102].

A major mechanism of antimicrobial resistance in the Bcc species is efflux pump-mediated extrusion, which has been associated with resistance to trimethoprim, chloramphenicol, tetracyclines, and some quinolone antibiotics [94,103,104,105]. For instance, the genome of the *B. cenocepacia* strain J2315 contains coding sequences for all five major families of efflux systems, including the RND superfamily, and 16 putative RND efflux pumps have been identified in this genome [106,107]. The RND-10 encoding *ceo* operon confers resistance to chloramphenicol, trimethoprim, and ciprofloxacin to all the previously susceptible *B. cenocepacia* isolates to which it was transferred, and deletion of the RND-3 and RND-4 operons results in increased sensitivity to aztreonam, chloramphenicol, gentamicin, and various quinolone compounds [96,108]. Recently, mutations in the RND-3 efflux pump regulator gene were correlated with heightened efflux pump expression levels in several clinical Bcc isolates, and accounted for the increased multidrug resistance of these strains, demonstrating the important contribution of efflux pumps to the Bcc resistome [109]. 

Antimicrobial agents to which the Bcc species currently remain at least somewhat susceptible include semisynthetic penicillins (such as ticarcillin), carbapenems (such as meropenem), cephalosporins (particularly ceftazidime), some quinolones (including ciprofloxacin) and, in some cases, trimethoprim/sulfamethoxazole [110]. Combination therapy has been proposed as a means of circumventing the development of problematic antibiotic resistance, and the combination of at least two, ideally three, antimicrobial agents is generally recommended for the treatment of Bcc-associated pulmonary exacerbations in CF patients [111]. In a recent study examining the safety and efficacy of treatments using meropenem and ceftazidime combined with tobramycin in CF patients, it was found that combinations of these drugs are generally well-tolerated, produce appreciable improvements in lung function, and significantly reduce bacterial density in patient sputum [112]. When investigating the potentially synergistic, additive or antagonistic in vitro effects of antibiotic combinations against a panel of 119 multidrug-resistant Bcc isolates, Aaron et al. found that although 50% and 8% of all isolates were resistant to all single-drug and two-drug treatments, respectively, all of the isolates were inhibited by combinations of three different compounds. Among double-antibiotic combinations, meropenem–minocycline, meropenem–amikacin, and meropenem–ceftazidime were found to have the strongest bactericidal effects, while combinations of tobramycin, meropenem and any other antibiotic were most effective among triple combinations [93]. More recently, a comprehensive study by Zhou et al., which tested 23 antibiotic combinations against a panel of over 2600 Bcc strains, found that while minocycline, meropenem and ceftazidime are the most efficacious overall, they nevertheless target only 38%, 26% and 23% of all strains, respectively [94]. Concordantly, the current treatment protocols for Bcc infections typically include tobramycin, trimethoprim–sulfamethoxazole, ceftazidime, meropenem, and minocycline, but resistance to these antimicrobials and combinations thereof is becoming increasingly common [113]. These findings underscore the challenging nature of managing infections with multidrug-resistant Bcc species in susceptible populations, and highlight the desperate need for novel modes of treatment. 

## 3. Bacteriophage Therapy

One of the alternative strategies proposed to combat multi-drug resistant bacteria is the therapeutic application of bacteriophages (phages). Phages are bacterial viruses—obligate intracellular parasites that infect, commandeer and destroy bacterial cells, in order to replicate themselves and subsequently exit the infected cells to acquire novel hosts. Given the inherently bactericidal nature of these microscopic replicators, their initial discovery and the conceptualization of their use as a means to combat bacterial infections are essentially inseparable. 

### 3.1. Lessons from History: Phage Therapy in the 19th and 20th Centuries

Although phage biologists often assert that the history of phage research begins with Twort and d’Herelle’s co-discovery in the early 20th century, phenomena caused by bacteriophage activity were reported as early as the late 19th century. Probably the oldest published account of a phage-related phenomenon is Ernest Hankin’s 1896 report on an antibacterial agent present in the Ganges river. Hankin found that the agent was able to pass through the finest available porcelain filters and continue to exhibit its antibacterial properties, but that this activity could be rapidly inactivated by even modest amounts of heat. Hankin noted that the residents of the nearby city of Agra considered the river to be holy, as the water exhibited healing properties—a phenomenon he considered to be related to the presence of the antibacterial agent [114]. Although poorly distributed at the time, similar research was conducted by the Russian biologist Nikolai Gamaleya, who described the lysis of *Bacillus anthracis* cells in samples of distilled water, and found that these water samples could subsequently be used to clear plates containing related strains of the bacterium [115]. Nearly two decades later, the English bacteriologist Frederick Twort published his seminal work on “transmissible bacterial lyses”. After having spotted *Staphylococcus*-containing Petri plates with filtered *Staphylococcus* lysate, Twort observed the formation of clear, ‘glassy’ patches, in which the bacteria seemed unable to grow, and found that this phenomenon reoccurs when the contents of these patches are passaged onto new plates containing *Staphylococcus* [116]. Similarly to Hankin, Twort found that the agent forming these patches could pass through fine filters and was deactivated by heat, but additionally discovered that the agent did not form patches in the absence of bacteria, nor in the presence of bacteria that had previously been killed by heat, and that it seemed to have no ill-effect on the general health of a number of inoculated animals. Although Twort is often recognized as a co-discoverer of bacteriophages, he was in fact skeptical that the patch-forming agent was an independent microbe, since it could not reproduce independently and caused no illness in animals, and instead hypothesized that the agent was a bactericidal enzyme, secreted by the bacteria themselves in order to eliminate competitors [116]. Similar experiments were carried out in Georgia by George Eliava, who commented on the bizarre disappearance of *Vibrio cholera* cells on plates treated with filtered *V. cholera* lysates, but these results were not published until later [117,118].

Although the discoveries of Twort and his predecessors certainly paved the way, it is scarcely disputable that the discoverer of bacteriophages was the French–Canadian microbiologist and polymath Felix d’Herelle [117]. Assigned to investigate an outbreak of hemorrhagic bacterial dysentery at a military encampment near Paris, d’Herelle—inspired by Twort’s work on bacterial lysates—made preparations from the stool samples of convalescent soldiers, and incubated them on plates containing *Shigella* strains that were isolated from these same patients. Unsurprisingly, d’Herelle noticed the appearance of “*taches vierges*” (clear patches), which he later called plaques, on the plates containing live *Shigella*, but not on the plates containing *Shigella* cells that had been heat-killed [119]. Unlike Twort, d’Herelle reasoned that since the agent formed plaques in discrete points on the plate, rather than being distributed throughout the plate uniformly, it was likely to be a microscopic virus rather than a secreted enzyme. These suspicions were later confirmed when d’Herelle found that although the agent was initially specific to a particular strain of *Shigella*, it became antagonistic against other strains after being added to a co-culture, followed by several passages in these new strains alone. Since enzymes and chemical compounds, unlike biological entities, are incapable of adapting in this way, d’Herelle had no doubt that the agent was an independent microbe, which he designated ‘bacteriophage’ (from ‘bacteria’ and Greek ‘*phagein*’ “to consume”, “to devour”)—a virus that exclusively destroys bacteria [119].

Realizing that the bactericidal properties of these microscopic replicators accounted for the convalescence of the patients from whom they had been recovered, d’Herelle immediately set out to discover whether these isolated bacteriophages could be used to rescue rabbits infected with a lethal dose of *Shigella*, and in doing this, he arguably founded the field of phage therapy [119,120]. Remarkably, all of the treated rabbits quickly recovered from *Shigella* infection, demonstrating the potential of phage therapy and prompting d’Herelle to pursue preliminary human trials with patients suffering from bacterial dysentery at the Hospital for Sick Children in Paris. The successes of these early trials led to the establishment of a laboratory in Paris, which was tasked with isolating and field-testing novel bacteriophages to be used to treat diseases such as cholera and plague, while other phage therapy pioneers in Western Europe extended d’Herelle’s initial discoveries to demonstrate that phages may be used to disinfect surgical wounds and treat *Staphylococcal* meningitis [117,121,122,123]. Importantly, d’Herelle and his Georgian colleague, George Eliava, conducted several expeditions to India, Southern China, and Southeast Asia, with the aim of combatting local cholera epidemics using isolated phages. In one longitudinal study utilizing phage to treat cholera, supervised by the British Army, the regions that accepted phage-mediated treatments reported mortality reductions as high as 90% relative to the regions that had refused bacteriophage treatments and had instead chose to rely on local folk medicines, such as plant extracts [117,121].

The reports of these successes transformed phage therapy into a lucrative industry, with several commercial laboratories and companies producing phage preparations in France, Germany, Brazil, and the United States [118,124,125,126]. For instance, by the early 1940s, the Indianapolis-based Eli Lily Company was mass-producing at least seven phage products to be utilized against a number of bacterial pathogens, including *Escherichia*, *Staphylococci*, and *Streptococci*, among others. These products consisted of filtered phage-lysed broths of target bacteria, and were used to treat a number of common infections, including abscesses, suppurating wounds, mastoid infections, vaginitis, and both acute and chronic infections of the upper respiratory tract [127]. Although these products proved useful in many experimental and clinical settings, their overall efficacy was a subject of great controversy, with several researchers reporting that phage products were unable to clear infections with consistency and reliability [128,129]. Although improper phage preparation techniques—resulting in low viable titers, due to phage inactivation—are likely to have contributed to these issues, reported inconsistencies in the efficacy of phage therapy are likely to have stemmed primarily from insufficient understanding of bacteriophage specificity, leading to physicians selecting incorrect phages for particular strains of target pathogens [127,130]. Because individual phages are often specific to particular strains of a given bacterium, and are incapable of affecting other strains, treatments utilizing phages require personalized strain identification and treatment, which is a strategy that was impossible given the technological capabilities available in the mid-20th century. Consequently, the therapeutic use of phage in mainstream western medicine was largely discontinued with the advent of broad-spectrum antibiotics, which were more reliable, had no specificity, and were much easier to dose and regulate, in the early 1940s—and the bacteriophage was subsequently relegated to the realm of the biological sciences [117,125]. 

Regardless of the increasing availability of broad-spectrum antibiotics in the 1940s, phages continued to be used therapeutically in Eastern Europe and the former Soviet Union, either as a complement or replacement of antibiotics, and several post-Soviet nations, including Russia, Poland, Georgia, Ukraine, Belarus, and Azerbaijan, continue to use phage therapy in some capacity today [118,127]. If Felix d’Herelle is to be considered the father of phage research in the west, his long-time Georgian colleague Georgia Eliava was certainly his Eastern European counterpart. Inspired by d’Herelle’s early successes, Eliava founded the Institute of Bacteriology (currently known as the Eliava Institute of Bacteriology, Microbiology and Virology; EIMBV) in Georgia, and collaborated with d’Herelle on a number of international projects that were intended to advance phage therapy. Although much of Eliava’s work was lost when he was executed as an ‘enemy of the people’ during Stalin’s purges of the 1930s, the EIBMV nevertheless remained active and produced the vast majority of the phage preparations that were utilized in Soviet experiments between 1940 and 1970 [118,127]. 

Expanding upon the pioneering work of d’Herelle and Eliava, Soviet researchers conducted many studies related to the phage-mediated treatment of bacillary dysentery, which, at the time, was a widespread and often life-threatening disease. Probably the largest study on this topic was conducted by Sapir, who described over one thousand human cases of both severe and non-severe bacillary dysentery that were treated with bacteriophages in Moscow. Interestingly, Sapir found that although phage treatment produced dramatic improvements in patient symptoms and significantly reduced the duration of hospital stays—reporting a 95% discharge rate after one week of treatment—he noted that this was not the case for chronically ill or terminal patients suffering from secondary infections, on whom these phage preparations had little effect [131]. Similar findings have been consistently reported by several other researchers, strongly suggesting that the use of phage exclusively as a treatment of last resort in terminally ill patients is inadvisable, since phages cannot target potential secondary infections, and the high fatality rate in these patients (regardless of treatment) artificially decreases the apparent success rate of phage therapy [9,127,132,133,134,135]. 

Although large-scale studies examining the prophylactic application of phage therapy are relatively scarce in western literature, prophylactic ‘phaging’ was carried out extensively in regions of the Soviet Union in which incidences of certain diseases were high [133]. Easily the largest and most thorough of these phaging campaigns was a year-long study conducted by the EIBMV, from 1963 to 1964, in which a polyvalent antidysentery cocktail, containing phages that were active against five major strains of *Shigella*, was administered prophylactically as a tablet to all children between 6 months and 7 years of age, on one side of every street in two Tbilisi districts, while the children on the other sides of the streets were given sugar tablets—thus forming a rigorous control group. After a year of monitoring over thirty thousand participants, researchers found that the bacillary dysentery rates were nearly four-fold higher in the untreated control group relative to the group treated with phage; a highly significant finding that demonstrates the massive antibacterial potential of phage therapy [9,136]. Comparably impressive prophylactic phaging campaigns were conducted by Soviet physicians, in response to cholera outbreaks in Pakistan (1958) and Afghanistan (1960), in which willing patients, who had already fallen ill, were treated with anti-cholera phage cocktails, while the susceptible populations were given phage tablets as prophylaxis. The reports from these campaigns suggest mortality rates of around 5% in the phage-treated groups, compared to over 50% in the patients who had volunteered to be treated with oxytetracycline and other antibiotics, which is comparable to the results obtained by d’Herelle in the 1920s and 1930s. As well, the incidence of cholera reportedly decreased rapidly following prophylactic phaging campaigns and disappeared completely in villages where the entire population accepted the phaging regimen, suggesting that phaging could be used to eliminate certain endemic diseases [9,118,137]. 

As the threat of clinically relevant antimicrobial resistance steadily increased throughout the 1980s and early 1990s, phage therapy entered its early renaissance period as researchers in both the Soviet Union and Western world slowly intensified their efforts to study phages as a means of combating the impending crisis [127,138]. Importantly, as previously disastrous diseases, such as dysentery and cholera, became rare in the developed world, phage therapy research largely shifted towards the treatment of nosocomial, opportunistic infections by pathogens such as those in the ESKAPE group (*Enterococcus faecium*, *Staphylococcus aureus*, *Klebsiella pneumoniae*, *Acinetobacter baumannii*, *P. aeruginosa*, *Enterobacter* spp.). Beginning in the early 1980s, Soviet research institutes published numerous studies demonstrating the effectiveness of phages against lung infections by *Staphylococcus* spp., *P. aeruginosa* infections in patients with cystic fibrosis, and eye, urinary tract, and surgical wound infections by a variety of microorganisms [9,117,118,127]. Particularly impressive in terms of both the scale and scope of their investigations was the lab of Stefan Slopek at the Hirszfeld Institute in Wroclaw, Poland, in which a number of large-scale phage therapy studies were conducted throughout the 1980s. In these studies, highly specific phages, targeting *Staphylococci*, *Escherichia*, *Klebsiella*, and *Salmonella*, were used to treat patients who had failed to respond to conventional antibiotics. Depending on the infectious agent in question and the duration of illness prior to treatment, the success rates in these trials reportedly ranged from 75 to 95%, clearly demonstrating the potential of phage therapy against these pathogens [9,127,139]. In the 1980s and 1990s, the phage therapy revolution slowly gained steam in Britain as well, with researchers such as Soothill demonstrating the ability of phages to consistently protect both mice and human skin grafts from deadly infections by *A. baumannii* and *P. aeruginosa*, thereby demonstrating the benefit phage therapy could have in treating infected surgical wounds [117,140,141]. Around this time, phage therapy also entered the arena of agriculture—with the goal of improving yields by protecting livestock from infection by species such as *Escherichia coli*. In the mid-1980s, a number of studies conducted by Smith and Huggins not only demonstrated that coliphage injection drastically reduces mortality in *Escherichia*-infected mice, calves, lambs and piglets relative to untreated controls, but also revealed that a single coliphage injection was more effective than multiple treatments using broad-spectrum antibiotics, such as tetracycline, streptomycin, ampicillin, or chloramphenicol. Interestingly, the authors noted that although phage-resistant mutants of *E. coli* were recovered from the stool samples of convalescent animals, these mutants were generally less virulent than their parent strains and caused minimal illness when re-inoculated into naïve animals [9,142,143]. Furthermore, follow-up studies demonstrated that when used prophylactically, coliphages produce equally significant reductions in mortality as when used therapeutically, suggesting that phages could be employed for both prevention and treatment in the agriculture industry [144]. 

As phage therapy research around the world became increasingly comprehensive in the early 1990s, several major advantages of phages over antibiotics became apparent, including the fact that they do not harm the commensal microbiome, their lack of cytotoxicity towards eukaryotic cells and, therefore, lack of side effects, and their ability to replicate at the infection site—often allowing infections to be cleared by a single therapeutic dose [140,145,146]. Encouraged by these discoveries and pushed onwards by the increasingly dire threat of antimicrobial resistance in the 2000s and 2010s, phage therapy research has fully entered its renaissance in the early 21st century—with researchers employing these microscopic replicators through a number of approaches designed to mitigate problematic phage resistance, including phage engineering, polyphage cocktails, phage–antibiotic synergy, and anti-virulence strategies, against a vast array of increasingly resistant and hazardous pathogens. 

### 3.2. Bacteriophage Lifestyle and Mechanisms of Infection

With an estimated 10^31^ phage particles in the biosphere, which is roughly 10-fold higher than the predicted number of bacteria, bacteriophages are widely recognized as the most abundant biological entities on the planet [6,7,147,148]. Phages are naturally present in all environments colonized by bacteria, including human tissues, and play a major role in bacterial ecological dynamics. Importantly, phage predation results in approximately 10^23^ infections per second and destroys an estimated 50% of the global bacterial population every 48 h, thereby contributing significantly to global carbon and nutrient flux [6,7,147,148,149,150]. From this perspective, phage therapy is simply the exploitation of a naturally occurring biocontrol system and its application to a human problem. 

Although bacteriophages, as all biological entities shaped by natural selection, exhibit great diversity in terms of structural and genomic organization, the non-enveloped, double-stranded DNA, tailed phages of the order *Caudovirales* are most relevant to the topic of phage therapy [151]. This order contains phages of the families *Myoviridae,* which possess long, rigid, contractile tails; *Siphoviridae*, whose tails are long and flexible, but have no contractile machinery; and *Podoviridae*, which have short, non-contractile tails [152]. These phages consist of a proteinaceous capsid composed of head and tail regions—the latter containing tail fibers, and a baseplate for reversible and irreversible receptor binding, respectively—and a linear double-stranded DNA genome, generally between 18 and 500 kilo base pairs in length, which is stored at high pressure in the head region [152,153,154]. 

**Figure 3:**
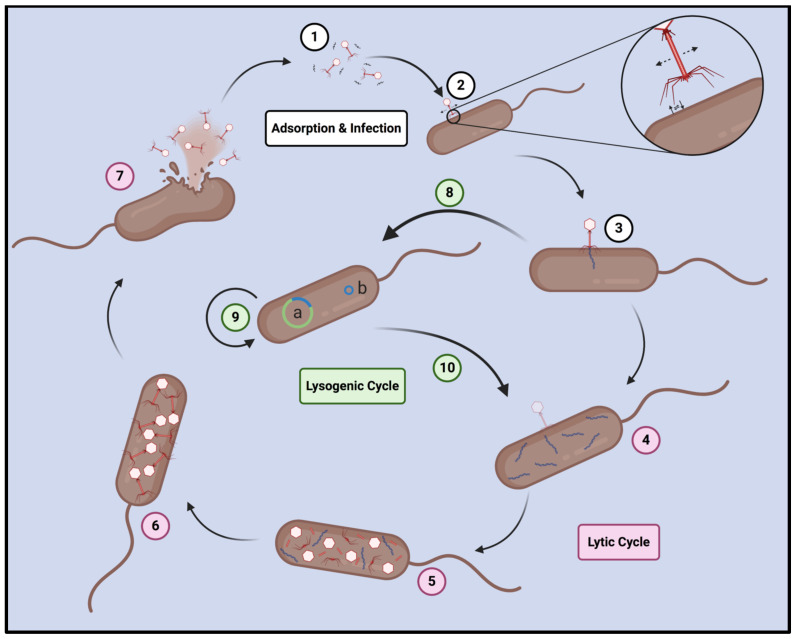
Replication cycle of *Caudovirales* bacteriophages. All phages of the order *Caudovirales* replicate through two complementary cycles. Prior to infection, phage particles are propelled through medium by random electrostatic interactions with nearby molecules (**1**) until they reach a bacterial host cell, whereupon they adsorb to its surface by using their tail fibers and subsequently utilize the spontaneous binding and unbinding of these tail fibers to engage in a random walk across the cell surface (**2**). Upon interacting with its cognate secondary receptor, the phage infects the cell by injecting its genetic material (**3**) and is then compelled to proceed with one of two possible replication cycles. In the lytic cycle, which is normally employed when host cell density is high, the phage genome is replicated (**4**), transcribed and translated into the components of the phage particles (**5**), which are then assembled within the cell (**6**). Finally, the host cell is lysed (**7**), and liberated progeny phages are able to subsequently infect additional cells. In the alternative lysogenic cycle, which is often employed when host cell density is low, the phage genome is either integrated into the bacterial genome (**8a**) or circularized into a phagemid (**8b**), and this prophage then forms replicates passively along with its lysogenized host (**9**). Deterioration of host cell conditions due to starvation or other stressors may cause induction of the prophage, whereupon it returns to the lytic cycle (**10**) and ultimately destroys its host cell via lysis.

The first step in the reproductive cycle of any bacteriophage is binding with its cognate receptor on the bacterial cell surface [155]. Unlike bacteria and higher lifeforms, phages do not possess the means to achieve self-directed motion, and they are instead propelled through a medium by the forces of random molecular bombardment—thus exhibiting Brownian motion (Figure 3). Since the odds of a phage particle interacting with its cognate receptor, while diffusing randomly through a three-dimensional medium, are infinitesimal, many phages utilize tail fibers, which bind reversibly to various structures (termed primary receptors) on the cellular surface [156,157]. Consequently, phages are able to use the arbitrary binding and unbinding of their tail fibers to engage in a random walk across the cell surface, in search of their true (secondary) receptor. Once this receptor has been reached, irreversible binding occurs between it and the phage’s baseplate receptor-binding proteins (RBPs), triggering a conformational change that opens the tail tube at both the proximal and distal ends, and allows the phage to inject its genome into the target cell [157,158]. Among *Myoviridae*, the energy derived from ATP hydrolysis drives the contraction of the tail, producing a motion akin to the depression of a syringe, through which the inner hollow tube of the tail punctures the outer cell membrane, and the genetic material within it is subsequently expelled from the capsid and inserted across the outer membrane [159,160]. Since *Sipho*- and *Podoviridae* lack contractile tails, capsid pressure is used to push the genome across the outer membrane [158]. In at least some cases, lysozymes or other viral enzymes that are attached to the baseplate are thought to facilitate genome insertion by degrading part of the outer membrane and cell wall, with fragments produced by this degradation acting as a signal to trigger the injection of the genome. Nevertheless, the precise mechanisms whereby phage genomes are trafficked through the cell wall and inner membrane remain poorly understood and are thought to vary widely among different phages [158]. Once the phage genome has reached the cytosol, environmental and host cell conditions direct lifestyle determination by the phage. 

Among phages of the order *Caudovirales*, two evolutionarily complementary lifestyles, termed the lytic and lysogenic cycles, are dominant [153,161]. In the lytic cycle, early gene products that are expressed from the injected genome are used to disrupt the normal bacterial processes, and begin the process of phage genome replication. Once a sufficient number of genomes has been produced, late genes are progressively expressed to hijack the bacterial ribosomes, and trigger the assembly and maturation of new phage particles, which are finally released from the host cell when it is lysed through the tightly regulated release of phage endolysins that destroy the cell membrane [161]. On average, a lytic cycle beginning with a single phage typically produces about 100 new phage particles in 25 to 30 min, meaning that the population of a lytic phage typically increases much more rapidly than that of its target bacterium. This inevitably results in the collapse of the bacterial population, which is followed by the collapse of the phage population, since the phage effectively runs out of viable hosts. In order to avoid this fate, many phages employ the lytic cycle only when the density of available hosts is high, but switch to the lysogenic cycle as the host density begins to drop [150]. In the lysogenic cycle, early and late lytic genes are repressed in favor of the expression of lysogenic genes, which trigger the integration of the phage genome into that of its host bacterium or, in the case of some phages, the circularization of the phage genome into a dormant plasmid, known as a phagemid [60,153]. Regardless of whether it is integrated or circularized, the dormant phage is known as a prophage, while the bacterial cell harboring the prophage is called a lysogen. The prophage subsequently replicates passively along with its lysogenized host, resulting in a population of lysogens [150,161]. Importantly, prophages may release ligands that interfere with the infection process of other phages that might infect the lysogen, or at least prevent them from entering their lytic cycle, leading to a form of phage resistance called superinfection immunity. This form of protection, however, is only effective against other virions of the same phage and perhaps other, very closely related phages, and is not a universal form of immunity [162,163]. Since the death of a lysogen results in destruction of the prophage it harbors, prophages are able to sense the viability of their host, in terms of nutrient availability and genetic integrity, and may revert to the lytic cycle, by reactivation of their lytic genes, if they sense their host will soon die [153,161,164]. In general, all true (non-satellite) phages are capable of undergoing the lytic cycle, while the possession of a lysogenic cassette is an evolutionarily beneficial, but ultimately non-essential, trait. 

Lytic phages have canonically been considered to be superior for phage therapy, with many contemporary authors arguing for the exclusive use of obligately lytic phages, since temperate phages possess a suite of characteristics that are problematic for therapeutic applications [165]. In particular, the fact that these phages do not rapidly eliminate all of their hosts suggests that they may be inefficient in clearing bacterial infections. As well, phage genomes may contain genes that increase antibiotic resistance or virulence when expressed in a lysogen, while superinfection immunity from the prophage may increase resistance to other phages, possibly producing an even more dangerous pathogen and exacerbating the infection [160,163,165]. Finally, the inserted genomes of temperate phages are also capable of transducing nearby bacterial genes, including those that are capable of increasing virulence or antibiotic resistance, to new host cells—thereby spreading these characteristics within or even between species [60,150]. Given the evolutionary benefits of being capable of lysogenization, however, it is reasonable to suspect that obligately lytic phages, which lack lysogenic genes altogether, may be the minority of all existing phages, and the exclusive use of such phages for therapy may therefore be an impractical approach [166,167]. Despite the issues associated with temperate phages, the effectiveness of such phages in both in vitro and in vivo therapeutic settings has been demonstrated by a number of authors [168,169,170,171,172], suggesting that at least some temperate phages are useful in therapeutic applications. 

### 3.3. Advantages of Phage Therapy Relative to Traditional Antibiotics

While limitations associated with phage therapy certainly exist, there are several major advantages to using bacteriophages rather than chemical antimicrobials when combating clinical bacterial infections, including unparalleled target specificity, a lack of cytotoxicity, environmental abundance, and unique pharmacokinetics.

Although the exquisite specificity that bacteriophages have for particular receptors (and therefore hosts) is frequently touted as a major advantage, the opponents of phage therapy have alternatively described it as a double-edged sword. Since a given bacteriophage requires a specific receptor, with a precise molecular structure at the RBP–receptor interface, in order to successfully infect and subsequently kill a target cell, bacterial species and strains that lack this receptor or possess sufficiently mutated versions thereof will be completely immune to infection [173,174]. Indeed, bacteriophages generally infect only 10–20% of all strains of a given bacterial species, and although polyvalent phages that are capable of infecting multiple bacterial species or even genera have been described, such phages are thought to be exceptionally rare [60,175,176,177]. One historically problematic consequence of this specificity is the need to isolate the particular strain of a pathogen that is causing illness in a given patient, and subsequently match this strain with a phage (or cocktail of phages) that is capable of destroying it in vivo. Since clinicians in the first half of the 20th century only possessed rudimentary means of discerning between bacterial strains and had no understanding of the specificity phages have for particular receptors, this requirement would have posed an insurmountable challenge at the time, and was indeed the primary cause for the eventual discontinuation of western phage therapy in the 1940s [117,127]. Given modern technological capabilities, however, the rapid isolation and identification of clinical strains is relatively straightforward, and the challenge has therefore shifted to the isolation of a sufficient diversity of phages, so as to target all the emerging clinical strains of a particular pathogen. This requires intensification of the efforts to isolate, characterize, and therapeutically validate novel bacteriophages, and the construction of a comprehensive and publicly available schedule of clinically available phages and their specific targets—an endeavor that is challenging, but certainly achievable, given technological abilities and the environmental abundance of phages. Although laborious, treatment personalization of this type has been proposed for certain onco- and immunotherapies, and is increasingly possible with improvements in laboratory automation and genomic technologies, meaning that personalized phage therapy may be utilized to circumvent the issue posed by bacteriophage target specificity in the near future [178,179]. Conversely, this exquisitely narrow specificity provides a major advantage, which cannot be boasted by even the most efficacious antibiotics. Since phages very rarely infect multiple distinct bacterial species, the risk of disrupting the commensal microbiome of the host as collateral damage is very low when compared to broad-spectrum antibiotics [8]. Indeed, while many chemical antimicrobials are known to promote superinfections, including *Candida albicans* yeast infections and *Clostridioides difficile* colitis, due to the well-documented dysbioses of the vaginal and gut microbiomes, respectively, no evidence of phage-associated superinfections has been reported as of yet, and several researchers have found that phage administration produces no significant alterations to the gut microbiomes of mice and livestock [180,181,182,183,184,185]. These results were corroborated in several studies involving healthy human volunteers receiving coliphage cocktails in Bangladesh, with authors consistently reporting no statistically significant long-lasting changes to patient microbiomes [186,187]. In a subsequent trial employing these cocktails to treat *E. coli* colitis, no substantial alterations to patient microbiomes were reported, but the cocktails also failed to produce appreciable effects against the target bacterium itself, which was likely due to strain mismatch—further highlighting the simultaneous advantages and drawbacks of phage target specificity [188]. Although further investigations are certainly necessary, these early findings suggest that phage therapy may improve patient well-being, relative to antibiotic therapy, at least partially due to the precise target specificity, which reduces the incidence of adverse effects that are triggered by disruptions of the normal microbiota.

The reduction in adverse effects associated with phage therapy is not limited to decreased disruption of the microbiome, however, and extends to a general lack of cytotoxicity towards the cells of the host. This is in stark contrast to the cytotoxic activity of chemical antibiotics, which are known to cause adverse effects ranging from fever and nausea to heart damage, immunosuppression, and major allergic reactions, including anaphylaxis, as well as diarrhea due to the disruption of the commensal microbiome [138,189]. Unlike pharmaceutical antimicrobials, which possess specific functional groups that are capable of interacting with target structures in host and bacterial cells alike, bacteriophages consist solely of capsid proteins and nucleic acid, and therefore have no ability to produce cytotoxicity directly. Since the cell-surface receptors that bacteriophages use to infect cells are unique to prokaryotes, phages have no means of entering eukaryotic host cells (other than via phagocytic internalization by the immune system; see Section 3.4), and are therefore cytotoxic only to the specific bacterial targets against which they are being deployed [8,190]. Empirical support for the lack of side effects associated with phage therapy has been available since the early experiments of d’Herelle, and has been found consistently by phage researchers well into the modern era [120,127]. One of the earliest and more comprehensive investigations into the therapeutic safety of phage in modern scientific literature was conducted in 2005 by Bruttin and Brussow, who administered coliphage T4 orally to healthy volunteers, in order to study the potential adverse effects. In addition to producing no appreciable alterations to the commensal microbiomes of these volunteers, the bacteriophage preparation resulted in no identifiable adverse effects, and no heightened serum transaminase levels—which would indicate liver toxicity—were detected. Furthermore, serum analysis found that no anti-T4 antibodies had been produced, demonstrating that T4 phage does not elicit an immune response [191]. Results similar to these have since been consistently reported in a plethora of studies testing bacteriophages against pathogens in various medical conditions, including bone and soft tissue infections [182,192], otitis [193], lung infections [135,194], disseminated infections [195,196], graft infections [197], prosthesis infections [198], burns [199], and diarrheal disease [186,187,188], thereby comprehensively demonstrating the superior safety and lack of adverse effects associated with phage therapy.

Another major advantage of phage therapy is that, unlike antibiotics, of which there is a relatively limited and largely exhausted repertoire, phages are environmentally abundant and exist as a population of an effectively unlimited and constantly increasing number of distinct types [4,7,150]. Indeed, a major driver of the phage therapy renaissance is the understanding that although novel compounds are increasingly needed as antimicrobial resistance gradually extends to all currently known drugs, the antibiotic pipeline is running dry, since the majority of naturally occurring clinically usable compounds seem to have already been discovered. Between 2003 and 2007, for instance, only five novel antibacterial drugs were approved for clinical use by the US Food and Drug Administration, and only two of these had novel mechanisms of action [4]. By contrast, a single investigation in 2007 found a total of 232 novel phage isolates that were capable of efficiently infecting over 70 different strains of *Salmonella*, demonstrating the massive and largely untapped reservoir of phages that could be exploited by an intensified effort of isolation and characterization [200]. Furthermore, because phages constantly adapt to changes in their bacterial targets, as part of an endless evolutionary arms race, the number of phage types is constantly increasing and there is no reason to suspect that there exists a bacterial species or strain for which a bacteriophage cannot be found [7,201,202]. This suggests that for any clinically relevant bacterial strain, phages that are capable of infecting and destroying it must exist, and the challenge for phage therapy is therefore their rapid isolation, characterization, and therapeutic validation. The primary sources of phages are the natural environments of their target bacteria, and they include soil, fresh and sea water, sewage, and the tissues of host organisms [7,147]. For clinically relevant bacterial strains, patient samples, such as the sputum and excrement of patients with respiratory and gastrointestinal infections, respectively, as well as relevant tissue samples, are established sources of bacteriophages. As was the case in d’Herelle’s initial discovery, freshly convalescent patients are likely to be the best source of phages for clinical strains, since naturally present phages that are capable of destroying those strains would have multiplied enormously while eliminating the infection, and could therefore be present in patient samples, but these phages might already have been eliminated in patients in the later stages of recovery [119,127]. One potential approach for isolating novel clinically useful phages is through artificial selection experiments, in which previously available bacteriophages and their target bacteria are applied to an animal model (or, theoretically, into a realistic in vitro analogue thereof), and novel phages that have evolved to infect the bacterial strains, which evolve under in vivo selection pressures, would subsequently be isolated [165,203]. One issue with this approach, however, is the potential difficulty of propagating such phages under normal laboratory conditions, since their bacterial targets might revert to their wild-type phenotype and become invulnerable to the evolved phages [194]. An alternative means of isolating novel temperate phages is by inducing prophages out of the lysogens of a given strain, and propagating the induced phage on an alternative strain that the phage is able to infect [204]. DNA cross-linking agents, such as mitomycin C, are capable of inducing prophages to the lytic cycle, thereby producing phage particles that can be collected, purified, and subsequently tested against other strains of the target bacterium. Temperate phages that have been isolated in this manner can be converted to obligately lytic variants through the inactivation or excision of their lytic repressor [166,196], or used directly through combination with other phages, to generate a polyphage cocktail [172,195]; both of which are approaches that have been used by a number of investigators to treat antibiotic-resistant infections in animal models, as well as in humans [167]. Lastly, novel phages that are capable of infecting an expanded or entirely distinct repertoire of hosts could be generated through the genetic modification of the receptor-binding proteins (RBPs) of existing phages. This approach was recently used to develop a massive library of slightly distinct phage types, which were subsequently used to completely eradicate *E. coli* populations in vitro [174]. 

Finally, the fact that phages are biological entities, rather than chemical compounds, provides them with an advantageous pharmacokinetic property. Specifically, phages are capable of replicating at the site of infection—exponentially increasing their numbers at the expense of their bacterial target—meaning that the contents of a single dose automatically increase their number, and remain viable at the site of infection for a substantial period of time. Importantly, this property means that a single dose of phage may be sufficient to eliminate an infection. This ability has been experimentally confirmed in countless animal models, since at least the 1980s [140,143,169], and has also been validated in a human phage therapy study by Wright et al., who demonstrated that a single dose of a topically applied polyphage cocktail was sufficient to resolve chronic otitis caused by *P. aeruginosa* [193]. Even in studies where a single dose is insufficient, the cumulative number of doses throughout the treatment period is quite low, ranging from one dose per month to one per week. This is in direct contrast with antibiotic therapy, in which the doses must be repeated frequently to maintain the titer, since chemical antimicrobials are incapable of replicating and must be supplemented at regular intervals [8,192,194]. Exceptions to this may occur when phages are used to target disseminated infections or other infections requiring phages to be administered intravenously, since phage half-life in blood appears to be limited, and phages must travel far to eliminate bacteria that are difficult to reach [205,206]. In these cases, treatment may last many weeks or even months, and typically requires a large number of repeated phage doses, often in combination with an arsenal of antibiotic compounds [135,195,196,198]. As well, since phages are applied to infection sites in large titers and replicate with short generation times, variant phages that are capable of infecting bacteria that have mutated to evade the wild-type phage may appear naturally and would be maintained by selection, thus preventing the formation of resistant outgrowths caused by slight mutational changes in the target bacteria. Co-evolution of this type is impossible with chemical antimicrobials, which are therefore quickly rendered ineffective by bacterial adaptation, thus underscoring the pharmacokinetic advantage of bacteriophages [207]. Ultimately, phage populations evolve rapidly in concert with their bacterial host, and maintain themselves at infection sites until the clearance of all the target bacteria or the appearance of a resistant outgrowth that a single phage cannot overcome. Although such phage-resistant outgrowths do appear during the course of treatment, several strategies have been developed to circumvent such problematic forms of resistance (see Section 3.5) [165,195,201,208,209,210].

Additional advantages of phage therapy potentially include the relatively low cost of developing and manufacturing phage-based medicines, phage versatility with respect to the forms of therapeutic application—including creams, pills, aerosols, and liquids for topical, oral, inhalatory and intravenous administration, respectively—and the relatively low impact that specific phages have on other bacteria in the environment. As well, phages do not interact directly with antibiotics or other pharmaceutical compounds, meaning that phages are unlikely to be a contraindication for certain patients, and may be used with relative universality [8,170]. Finally, unlike chemical antimicrobials, many phages are able to penetrate into and destroy biofilms, possibly due to the expression of exopolymer-degrading depolymerases, but this phenomenon remains incompletely understood and warrants further investigation [9,211,212,213].

### 3.4. Interactions between Bacteriophages and the Mammalian Immune System

**Figure 4:**
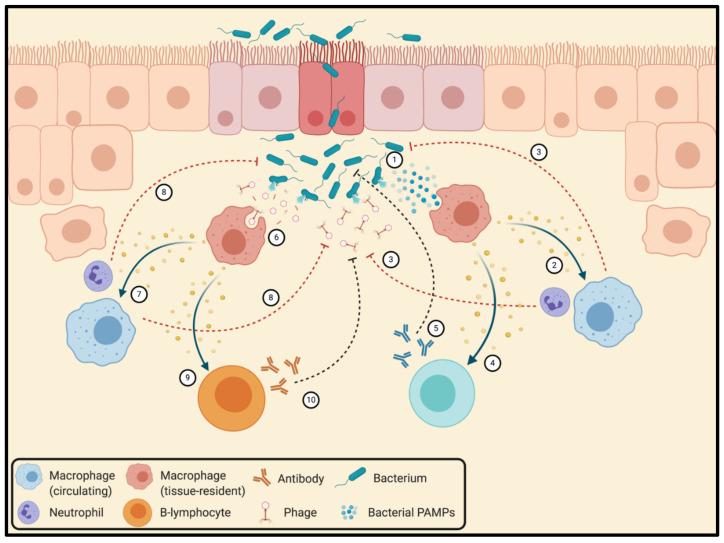
Interactions between bacteriophages and the mammalian immune system. In addition to their direct anti-bacterial action via killing of target bacterial cells, phages can contribute to bacterial killing indirectly by stimulating the host immune system—a multifactorial process that can be divided into two main pathways. At the infection site, phage-mediated lysis of bacteria induces the release of bacterial pathogen-associated molecular patterns (b-PAMPs), which trigger the activation of tissue-resident macrophages (**1**). Upon activation, these macrophages use cytokine signaling to activate circulating macrophages and neutrophils and recruit them to the infection site (**2**), where they indiscriminately eliminate phages and bacteria alike via phagocytosis followed by ROS-mediated degradation (**3**). Furthermore, cytokine signaling by b-PAMP-activated tissue-resident macrophages leads to the activation and recruitment of B-lymphocytes (**4**), which subsequently initiate a specific humoral response against invading bacteria (**5**). Simultaneously, phage particles (and fragments thereof) introduced through treatment and reproduced via the lytic cycle stimulate the activation of other tissue-resident macrophages (**6**), which similarly recruit circulating macrophages and neutrophils (**7**) that subsequently eliminate both bacteria and phages via indiscriminate internalization and degradation (**8**). Finally, tissue-resident macrophages may activate circulating B cells through presentation of phage peptides (**9**), leading to a specific humoral response against phage particles (**10**).

Interactions between bacteriophages and the innate and adaptive immune systems of higher mammals have been studied since at least the 1960s, and there remains little debate today about the fact that bacteriophages are recognized by immune cells. Nevertheless, the extent to which the immune recognition of phages helps or hinders therapeutic outcomes remains poorly understood, and a universal understanding of phage–immune dynamics remains elusive, since immunostimulatory effects appear to vary widely between different phages [214]. Generally speaking, phage–immune interactions can be classified as those that stimulate responses against the phage itself, and those that stimulate responses against invading pathogens (Figure 4). 

It has long been known that phage recognition and endocytosis by phagocytes, such as macrophages and neutrophils, occur in a time-, concentration-, and temperature-dependent manner, and contribute enormously to the elimination of phages from the body [215]. This phenomenon has been described in detail for several well-characterized phages, including T4, λ, P22, and φX174, which are cleared via tissue-resident macrophage-mediated endocytosis from several organs, notably including the liver and spleen [216]. During phage–phagocyte interactions and the subsequent internalization, the binding of bacteriophage peptides and genetic material to pathogen-recognition receptors (PRRs) triggers the production of various cyto- and chemokines, which activate and recruit other immune cells to the site of infection. Immune activation of this type can also occur through the phage-mediated lysis of target bacteria, which releases new phages along with capsid proteins and naked phage DNA (collectively referred to as phage ‘pathogen’-associated molecular patterns, p-PAMPs). Crucially, phagocytes recruited to the infection site through phage stimulation do not discriminate between phages and bacterial pathogens—meaning that phage-mediated immune activation can assist in combating bacterial infections [214]. Interestingly, however, several murine studies have found that neither whole phages nor parts of their capsids appear to elicit cytokine production, possibly suggesting that immune activation is not triggered in this way by all phages [168,217,218]. Stimulation of this type ultimately leads to adaptive immune activation and, although there is no evidence of direct T-cell responses to phages yet, B-cell activation and production of anti-phage antibodies is well characterized for a number of phages, including φX174, which is often used in diagnostic tests for antibody response in patients with hypogammaglobulinemia or HIV [214]. Indeed, antibody induction against phages is likely part of the normal mechanism for removing phages from the body, since phage titers in SCID and B-cell-deficient mice, as well as human patients with X-linked agammaglobulinemia, decrease at greatly reduced rates relative to those seen in healthy controls [219,220]. Although at least one recent murine study found that induction of anti-phage IgG, and, to a much lesser extent, IgM, greatly reduces phage titers in the bloodstream and several organs, the authors noted that the effects on phage therapy of acute infections are likely to be minimal, since the infection should be cleared by the time of IgG induction against the phage [214,221]. Indeed, while the eventual induction of anti-phage antibodies is likely unavoidable, whether or not this affects the therapeutic outcome is not well understood, and likely differs with individual phages and their mechanism of delivery. Although much work remains to be conducted, several preliminary human studies have shown that the rates of antibody-mediated phage inactivation are low, especially when phages are delivered orally, and that there is little correlation between increased antibody titer and therapeutic failure [222,223,224]. Nevertheless, the induction of strong phage-neutralizing antibodies may be problematic for the phage therapy of chronic or recurrent infections, and the construction of a population of related phages that differ slightly in their antigenic regions, similar to that recently developed by Yehl et al., may therefore be advisable for resolving this issue [174,214,221].

In addition to their direct antibacterial activity through the lysis of target cells, phages may increase bacterial killing through stimulation of the immune system against invading pathogens, a phenomenon that can occur through two mechanisms. First, the direct activation of tissue-resident macrophages by phages leads to the recruitment of other phagocytes, including neutrophils, which indiscriminately internalize and destroy both the phages and bacteria at the site of infection. The in vivo reduction of phage titer might therefore be due to increased internalization by the recruited phagocytes, which simultaneously destroys the pathogens, meaning that apparent phage instability in vivo does not necessarily constitute therapeutic failure. Secondly, and most importantly, phage-mediated lysis results in the release of highly immunostimulatory bacterial PAMPs (e.g., endotoxin), which activate both innate and adaptive responses to combat the bacterial infection. Through these mechanisms, phages interact synergistically with the immune system to eliminate the infection. This paradigm was convincingly demonstrated in a recent seminal study by Roach et al., who demonstrated that while *P. aeruginosa* infection was fatal in both saline-treated immunocompetent and phage-treated neutropenic mice, the phage-treated immunocompetent mice had low bacterial titers and could be rescued from infection. Furthermore, RAG2^-/-^ Il2rg^-/-^ mice, who had no adaptive immune response, were also rescued from infection, suggesting that the innate immune response alone plays a major role in what Roach et al. dubbed immunophage synergy [218].

### 3.5. Strategies for the Circumvention of Problematic Phage Resistance

In order to avoid the failures that have resulted in the current antimicrobial resistance crisis, phage therapy must be deployed through various strategies that are designed to circumvent or mitigate the development of clinically problematic phage resistance. More theoretically grounded and scientifically rigorous than the relatively unguided approaches used by d’Herelle and his contemporaries, these strategies represent the second generation of phage therapy—sometimes referred to as phage therapy 2.0 [225]. Central to these strategies is the understanding that regardless of the level of sophistication of a given antibacterial treatment, mutants that are resistant to it will inevitably emerge, due to the large sizes and short generation times of bacterial populations [167,210]. Consequently, the goal of a therapeutic strategy should not be to eliminate the bacterial population entirely, but to greatly reduce its size, prevent resistant outgrowths, and select against virulence—with the ultimate aim of generating a small (and ideally avirulent) population, which the immune system, even if partially compromised, is able to eliminate [165,167,201,218].

**Figure 5:**
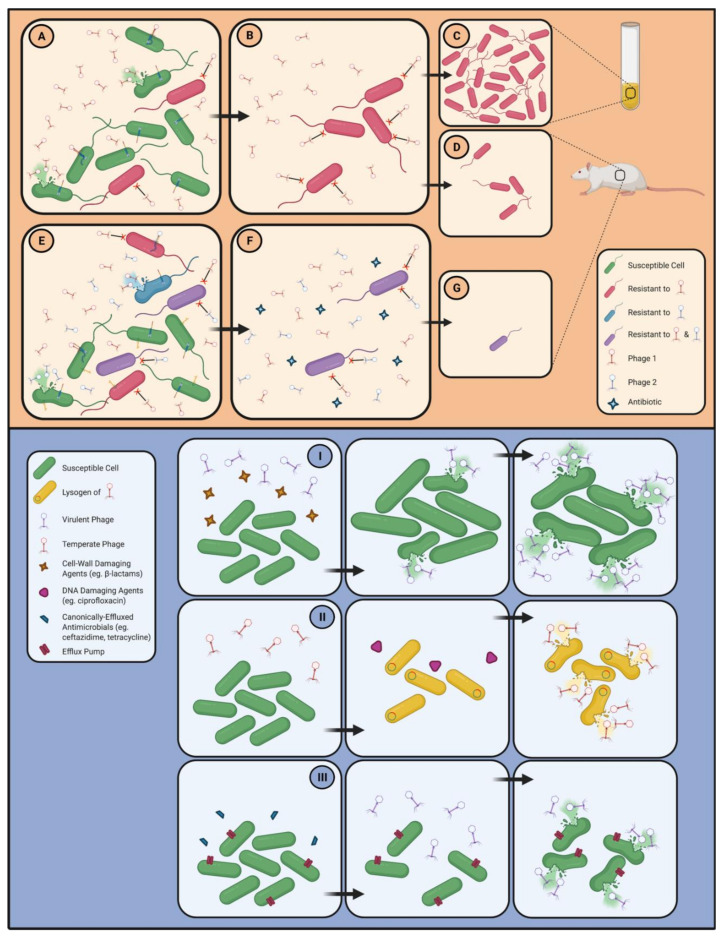
Phage therapy 2.0. Top panel: strategies for the circumvention of problematic phage resistance. One major therapeutic approach is the "*Anti-virulence strategy"*, in which phages are utilized as a selection pressure favoring avirulent bacterial phenotypes. To achieve this, clinicians may employ phages that utilize virulence structures, such as pili, as primary receptors (**A**). When such a phage is employed, susceptible cells are destroyed and the survivors, although invulnerable to infection, lack critical virulence factors (**B**). These avirulent survivors (red) may grow normally in vitro (**C**), but are unable to evade the host immune system in vivo and therefore grow poorly (**D**). Targeting a single virulence factor may be insufficient to eliminate in vivo virulence and thus survival, however, and the *Anti-virulence strategy* should therefore be combined with the *Multiple-targets strategy*, in which a cocktail of at least two phages—which target distinct virulence factors as receptors—is employed (E). Such treatments leave a small number of survivors, including rare mutants resistant to both phages (**F**). By combining this treatment with an antibiotic regimen, to create a polyphage–antibiotic cocktail, clinicians can reduce the bacterial population even further—to a point at which the host immune system, even if partially compromised, is able to eliminate the infection (**G**). Bottom panel: mechanisms of phage–antibiotic synergy. Although polyphage–antibiotic cocktails are efficacious even when the effects of their constituents are merely additive, several combinations are now known to produce synergistic killing effects through at least three putative mechanisms of action. In the *Delayed lysis mechanism* (**I**), co-treatment with phage and subinhibitory doses of antibiotics that target the bacterial cell wall, such as β-lactams, causes swelling and elongation of effected cells. Although this delays the time to lysis, it causes an increased buildup of phage particles inside the cell leading to increased burst size, which can allow for more rapid overall killing of the bacterial population. In the *Induction mechanism* (**II**), treatment with a temperate phage is followed by treatment with a DNA-damaging agent, such as ciprofloxacin, which induces the prophage to the lytic cycle. Since most of the survivors of the initial phage attack are lysogens, their induction to the lytic cycle destroys the vast majority of the overall bacterial population. Similar to the *Anti-virulence strategy*, the *Phage–antibiotic catch-22 mechanism* (**III**) utilizes a particular combination of phage and antibiotics as opposing selection pressures. Treatment with tetracycline, for instance, against which a major mechanism of resistance is efflux, selects for a bacterial population that possesses the efflux pump. Subsequent treatment with a virulent phage utilizing the efflux pump as a receptor therefore allows most of the population to be infected and eliminated.

Bacterial resistance to phages (phage resistance) can occur through a wide array of distinct mechanisms, including the loss or mutation of a receptor, receptor masking, production of ligands that compete with phages for receptor-binding, superinfection immunity, abortive infection systems, restriction modification systems, as well as CRISPRs and other forms of bacterial adaptive immunity [162,201,203,226]. Although this list seems daunting from a therapeutic perspective and is indeed largely fatal to the concept of monophage therapy, phages in nature nevertheless manage to propagate and are in fact reported to destroy roughly half of global bacterial populations every 48 h [6,7,148]. This ability to overcome bacterial phage resistance stems partially from phage-encoded countermeasures, but is largely due to the fact that phage populations are extremely and increasingly diverse, meaning that bacteria are constantly bombarded by a vast array of different phage types, and are unable to simultaneously adapt to all of them [7,162,201]. Furthermore, bacterial populations seeking to escape phage predation often participate in evolutionary trade-offs, which limit their ability to thrive in particular environments, leading to their elimination through other means [227,228]. These observations have led to the development of the following two main complementary therapeutic strategies: the anti-virulence strategy and the multiple-targets strategy (Figure 5, top panel); the latter of which can be achieved through polyphage or phage–antibiotic combinations. 

#### 3.5.1. The Anti-Virulence Strategy

Fundamentally, the anti-virulence strategy is the use of a bacteriophage as a selective pressure that shifts the bacterial population to one that carries fewer virulence factors, and is therefore less adept at colonizing its host and evading the immune system. Bacterial surface structures such as flagella, pili, adhesins, and iron transport proteins, which are essential for host colonization, as well as LPS, which is heavily implicated in immune evasion, also serve as primary receptors for many bacteriophages [158,173,229]. If phages that are capable of infecting through these targets are utilized, mutant bacteria in which these structures are absent or under expressed will survive and dominate, giving rise to a population which, although invulnerable to the administered phage, will have an attenuated ability to colonize, survive, and cause disease in the host organism [165,201,226]. Although its development into a therapeutic strategy is relatively recent, the phenomenon of anti-virulence selection has been understood by phage therapy researchers at least since the pioneering experiments of Smith and Huggins in the early 1980s. In these studies, the *E. coli* survivors that were isolated from coliphage-treated mice, calves and piglets were shown to have attenuated virulence, as they were unable to cause disease when re-inoculated into naïve animals [142,143]. Similar results were obtained in studies investigating the phage treatment of murine infections by *Yersinia pestis* [230], *A. baumannii* [203] and *P. aeruginosa* [231], with authors consistently describing bacterial survivors that were able to grow normally in vitro, but had an attenuated or entirely abrogated ability to cause persistent infections in vivo [226]. Additionally, this phenomenon has recently been confirmed in at least one case study of human phage therapy, where phage-resistant *A. baumannii* survivors, isolated from a convalescent phage-treated patient, had the same capsule-negative morphology that was correlated to an avirulent phenotype in a previous study [195,203]. Since the loss of a single virulence factor through selective pressure by monophage therapy may reduce, but not entirely eliminate, in vivo virulence, the anti-virulence strategy ought to be combined with the multiple-targets strategy, by assembling a polyphage cocktail in which each constituent phage infects through a distinct virulence-related surface structure, thereby maximizing the anti-virulence effect, while simultaneously reducing the likelihood of the emergence of a resistant outgrowth. A related approach is to utilize phages to target the virulence-like factor of antibiotic resistance—a strategy that is discussed at length in Section 3.5.3. 

#### 3.5.2. Polyphage Cocktails

Generally speaking, as the number of phages targeting distinct receptors in a given polyphage cocktail increases, the probability of the appearance of an outgrowth that is simultaneously resistant to *all* of these phages decreases rapidly, regardless of the nature of the receptors that the phages target. All therapeutic and agricultural applications of phages should therefore ideally employ polyphage cocktails with as large a number of phages as possible, in order to reduce the risk of the occurrence of phage resistance [165,178,201,232]. This principle is well established in modern phage therapy, since it is identical to the one that has been used to guide the successful development of chemical antimicrobial and antiviral cocktails since at least the late 1980s [232,233]. Consequently, the field is rife with phage therapy trials employing polyphage cocktails, including several successful human trials, with authors consistently reporting the predictable superiority of these cocktails over monophage therapy [135,172,182,187,193,195,196,213,234,235,236,237,238]. Despite the criticisms levelled against temperate phages by a number of authors, polyphage cocktails that combine temperate phages with more virulent types also appear to be highly effective [165,172,187]. 

#### 3.5.3. Phage–Antibiotic Cocktails and Phage–Antibiotic Synergy

Although phage–antibiotic cocktails and polyphage cocktails are both supported by the same basic concept of attacking multiple distinct targets simultaneously, there is, at least theoretically, a greater likelihood of finding a phage–antibiotic pair that satisfies this requirement, since many chemical antimicrobials target intracellular structures that are unlikely to be affected by the selective pressure of phage predation. In this sense, antibiotics can be considered a complement, rather than competitor, of bacteriophages [8,210,226]. Phage–antibiotic combinations are thought to be especially promising with regards to the elimination of biofilms, because while antibiotic penetration into biofilms is generally poor, phages expressing exopolymer-degrading depolymerases may be capable of destroying the outer barriers of biofilms, and subsequently allowing both phage- and antibiotic-mediated killing of target bacteria within [210,212]. Although phage–biofilm interactions remain incompletely understood for most species, a number of preliminary studies conducted over the past decade demonstrate the impressive ability of phage–antibiotic cocktails to degrade both mono- and poly-species biofilms [211,213,239,240,241]. Phage–antibiotic combinations may have additive, synergistic, or antagonistic effects, which is a phenomenon that varies significantly with different antibiotics, and appears to depend greatly on both the concentration of the antibiotic and the order in which the two components are administered [226,240,241]. 

Phage–antibiotic synergy (PAS) was first described for bacteriophages infecting certain strains of *E. coli*, as researchers noticed a significant increase in phage plaque size in the presence of subinhibitory concentrations of certain antibiotics, and this phenomenon was subsequently confirmed in *S. aureus*, *B. cenocepacia*, and *P. aeruginosa* [242,243,244,245,246]. While this plaque-size based approach may still be used to screen phage–antibiotic pairs for potential synergies, the therapeutic applicability of phage–antibiotic combinations exhibiting synergy is now more commonly verified through liquid-growth assays, where growth reductions greater than the sum of the independent growth reductions of the constituent parts may indicate a synergistic effect [239,240,241,247]. Impressively, synergistic interactions between polyphage cocktails and antibiotics were recently shown to fully resolve murine *P. aeruginosa* endocarditis and systemic *A. baumannii* infection, as well as *P. aeruginosa* aortic graft infection and disseminated *A. baumannii* infection in human patients, demonstrating the great potential of this form of combined therapy [195,197,231,248]. 

At least three mechanisms have recently been proposed to explain phage–antibiotic synergy on a cellular level (Figure 5, bottom panel), two of which rely on antibiotic-mediated stimulation of the bacterial stress response [164,249,250]. Probably the best studied of these is the ‘Delayed Lysis Mechanism’, in which antibiotic-mediated induction of the stress response causes overexpression of *RecA*, which, in turn, causes several metabolic and physiological changes, including, importantly, cellular swelling and elongation in Gram-positive and Gram-negative organisms, respectively. Due to this increase in cellular surface area, normal holin concentrations are insufficient to trigger phage release, and lysis is therefore delayed until a sufficient amount of holin has been produced. As a result of this delayed time to lysis, more phages are assembled within the cell prior to lysis, thereby increasing the burst size and facilitating the more rapid elimination of bacteria [243,244,250]. Interestingly, morphological changes resulting in delayed lysis time occur in *RecA* deletion mutants as well, suggesting that alternative pathways leading to swelling and filamentation phenotypes must be present [250]. In the related ‘Induction Mechanism’, which is only applicable to combinations involving phages that are capable of lysogeny, antibiotic-mediated stimulation of the bacterial stress response similarly results in the overexpression of *RecA*, which subsequently induces prophages to the lytic cycle. Temperate phages and stress-inducing antibiotics, such as ciprofloxacin, can therefore be potently synergistic combinations, since the phage will destroy at least some bacterial targets directly, while lysogenized cells will subsequently be destroyed from within, by induction of their prophages [164,249,251]. Importantly, the temperate phages used in such combinations may also have increased the lysis times, and thus burst sizes due to the morphological changes in the targeted bacteria, meaning that the antibacterial effects of the ‘Delayed Lysis’ and ‘Induction’ mechanisms can be challenging to separate when temperate phages are being utilized. A third mechanism, which could potentially be referred to as the ‘Phage–Antibiotic Catch-22′, relies on the strategy of using specific bacteriophages and antibiotics as oppositely acting selection pressures, which forces bacterial populations into unwinnable scenarios [209,210,226]. The best example of this approach was recently described by Chan et al., who utilized the *myovirus* OMKO1—which infects *P. aeruginosa* through an essential component of the multidrug efflux pump—as a selection pressure, to shift the bacterial population to a state that lacks this resistance structure, and is thereby rendered susceptible to antimicrobials against which the main form of resistance is extrusion. Since resistance to OMKO1, via decreased expression of the efflux pump, renders the bacterium susceptible to antibiotics, while resistance to antibiotics causes susceptibility to the phage, the bacterial population is unable to adapt to both of these opposing selection pressures and is more effectively eradicated. This bacteriophage was subsequently used in concert with ceftazidime, a compound against which efflux is a common form of resistance, to completely eliminate an aortic graft infection by *P. aeruginosa* in a human patient [197,209]. A more recent study by Gordillo-Altamirano et al. similarly demonstrated that treatment with capsule-binding bacteriophages selects for capsule loss in several clinical isolates of *A. baumannii*, and thereby resensitizes these strains to several previously ineffective antibiotics, as well as human complement, providing yet another example of the use of phages as a selection pressure that operates synergistically with antibiotics [248]. Similar results were recently reported in other human trials, in which the use of polyphage cocktails to treat *P. aeruginosa* bronchiectasis and disseminated *A. baumannii* infection appeared to sensitize the remaining bacterial populations to treatment with antibiotics, thereby indicating that this mechanism of exploiting evolutionary trade-offs may be a viable approach to formulating polyphage–antibiotic cocktails [135,195,226].

## 4. Bacteriophage Therapy Targeting the *Burkholderia cepacia* Complex

Although phage therapy has been used against various pathogens for over a century, the use of bacteriophages to specifically eliminate Bcc infections is a relatively novel endeavor, which began about two decades ago with the discovery of the first Bcc-targeting phages [252]. While phage therapy is not necessarily advisable for all species of bacteria—since some species can be targeted through other means, or are difficult to target using bacteriophages—the therapeutic use of bacteriophages against the Bcc species is advantageous and highly recommended for several reasons [253,254]. First, both CF and CGD patients, the main Bcc-susceptible populations, are treated with high dosages of multiple antibiotics from early ages, in order to prevent and suppress persistent infections, which greatly contributes to the evolution of a population of multidrug-resistant bacteria [10]. Since the efficacy of bacteriophage therapy is unaffected by antibiotic resistance, phages serve as an entirely independent mode of treatment, and patients afflicted by Bcc infections should therefore be treated with polyphage–antibiotic cocktails *ab initio*, in order to simultaneously minimize the evolution of both antibiotic and phage resistance. Next, Bcc-afflicted CF patients are regularly tested at CF clinics, and the strains from their lungs are routinely isolated for future reference, meaning that these strains could easily be screened for phage sensitivity, with little need for additional patient involvement. Because CF patients are usually afflicted chronically by a single Bcc strain, with little replacement by other strains, this approach can be employed to identify phages that could subsequently be used therapeutically in the patient [75]. As well, although the lungs of CF and CGD patients often harbor a wide variety of bacterial species, which exacerbate respiratory illness, the Bcc species are known to contribute heavily to this illness (e.g., via *cepacia* syndrome), and their specific elimination often greatly reduces morbidity and mortality rates in these patients [71]. Finally, the Bcc belong to one of many bacterial genera that are not defended by the CRISPR defense systems that may render phage therapy less effective, and so the Bcc may theoretically be more susceptible to this mode of treatment. This CRISPR deficiency is at least partially compensated for by other forms of phage defense, and the fact that the Bcc genomes are typically littered with both intact and deficient prophage sequences, which may encode ligands that interfere with the infection process of other phages via superinfection immunity [10,175,255]. Nevertheless, superinfection immunity is known to affect only those phages that have a certain degree of similarity to the endogenous prophage, and it is not a universal form of immunity [150,163], indicating that although the Bcc are highly resistant to chemical antimicrobials, their level of phage resistance may be limited. 

### 4.1. Bacteriophages Targeting the Bcc

Relative to other pathogenic bacterial species, against which hundreds of phages have been isolated and characterized, the Bcc appear to have a substantially smaller predatory phage population, and a comparatively small number of Bcc-targeting bacteriophages have been fully isolated since the inception of research into Bcc-specific phage therapy. As of 2021, only thirty-four of these phages have been reasonably well described in the scientific literature, and they are discussed in this review (Table 1). Bcc phages have been isolated from various environmental sources, including sewage, pond sediment, and the rhizospheres of onion, corn, and several other plants, as well as through induction of prophages and the isolation of spontaneous mutants of previously characterized phages [175,255,256,257]. Unusual among the general *Caudovirales* phage population, which is dominated by *siphoviridae*, the known Bcc phage population consists mainly of *myoviridae* (24 phages; 70%), while the Bcc *sipho*- (6 phages; 18%) and *podoviridae* (4 phages; 12%) are greatly outnumbered. The Bcc phages vary greatly in terms of the size and composition of their host ranges, with phages such as KL3, Bcep1 and Bcep22 each infecting only a single known Bcc strain, whereas the host ranges of other phages are more diverse—an example being the *myovirus* NS2, which is capable of infecting over twenty strains within four distinct Bcc species [7,60,255,256,258]. Interestingly, Bcc *myoviridae* typically have the widest host ranges, while the host ranges of *sipho*- and *podoviridae* are relatively small, although this finding might be an artefact of the currently small sample sizes of the latter two families. Five of the known Bcc phages are obligately lytic and the lifestyles of another eight are uncertain, due to lack of sequencing, whereas over half of the Bcc phages are known to be capable of lysogeny. Curiously, all four Bcc-podoviridae, three of which exhibit genetic relatedness, are either obligately lytic or form unstable and short-lived lysogens. Crucially, the capability of many of these phages to form lysogens has done little to inhibit their antibacterial effects, with lysogeny-capable phages, such as KS14 and KS4-M, demonstrating remarkable effectiveness in both in vitro and in vivo settings [166,170,244,256,259]. Interestingly, several Bcc phages have been isolated from the Bcc strains that they lysogenize, but these strains nevertheless remain susceptible to several other Bcc phages—suggesting that although prophages may provide superinfection immunity against phages of the same type, they do not provide protection against all Bcc phages [175,252,259,260,261,262]. In spite of over two decades of research, very little is known about the receptors that Bcc phages use to enter their target cells, with only the primary receptors known for a small number of phages. Indeed, LPS remains the only known primary receptor for Bcc phages, with JG068, ϕH111-1 and KS5 binding to the inner core, and KS9 binding to a motif distal to lipid A, while the specific LPS binding targets of KS12, NS1 and NS2 remain unknown. Additionally, it is known that the *siphoviridae* KL1 and AH2 do not bind LPS, but their primary receptor has yet to be elucidated [60,166,252,257,259,261]. 

Probably the most widely known of the Bcc bacteriophages is the *myovirus* KS4, a prophage of the *B. cenocepacia* type strain J2315, which was independently discovered as DK4 by Langley et al., as BcepMu by Summer et al., and as KS4 by Seed and Dennis [175,176,255,263]. Importantly, the latter authors found that although KS4 is unstable, has a limited host range, and is unsuitable for therapeutic applications, a spontaneously occurring mutant thereof, dubbed KS4-M, is able to efficaciously target additional pathogenic strains of *B. cenocepacia* and *B. multivorans*. Subsequent studies have found that KS4-M can be purified and processed into lyophilized and aqueous forms, which can be used for both intraperitoneal and inhalatory phage delivery, and to reduce mortality rates and bacterial burden in experimental in vivo models of phage therapy [169,170,175,264]. 

Other well-studied Bcc phages include the *podoviridae* JG068 and BcepIL02, and the *myoviridae* KS5, KS12, KS14 and AP3, which have been shown to reduce mortality rates in vivo, and the *siphovirus* KS9—which, in addition to in vivo effectiveness, is the only Bcc phage to have been converted to an obligately lytic variant (KS9c) by insertional inactivation of the gene encoding its lytic repressor [166,168,169,170,259,265]. As well as this, both KS12 and KS14 interact synergistically with a number of antibiotics in both in vitro and in vivo settings [244].

### 4.2. Bcc Phage Therapy 2.0

Although no studies investigating the anti-virulence strategy against the Bcc species have been conducted as of yet, this approach is considered to be a promising avenue for Bcc phage therapy, due to the abundance of cell-surface virulence factor structures among these species. Bacterial lipopolysaccharide (LPS), which is the primary receptor for a number of Bcc phages, is an important component of the Gram-negative bacterial cell surface, and plays major roles in both inflammation and immunomodulation in a number of species [252,259]. The structure of LPS, which typically consists of an O-antigen polysaccharide, core oligosaccharide, and lipid A, is heavily modified in Bcc species, through inclusion of the unusual sugar D-glycero-α-D-talo-oct-2-ulopyranosylonic acid (KO) in the inner core oligosaccharide, as well as the 4-amino-4-deoxyarabinose (Ara4N) residues bound to the phosphates of lipid A. These modifications are known to reduce the anionic charge of the Bcc cell surface, and thereby inhibit the binding and subsequent effects of cationic antimicrobial peptides [101]. The LPS of the Bcc species is highly inflammatory, causing significant upregulation of the pro-inflammatory cytokines IL-6 and IL-8, as well as TNF-α, and is able to activate immune cells through signaling via TLR4 [266,267]. The Bcc LPS has a toxicity that is approximately five-fold higher than that of *P. aeruginosa*, and it is believed to contribute greatly to the high level of inflammation associated with Bcc infections [268]. Therapeutic application of phages might therefore reduce Bcc-associated mortality rates, by using LPS-binding bacteriophages as a selection pressure to push infecting Bcc populations to a state with reduced inflammatory and cationic peptide resistance capabilities.

**Table 1:** Phages of the *Burkholderia cepacia* complex (Bcc) *.

	Phage Name ^†^	Morphology	Genus (Closest Relative)	Source	Bcc Host Range [*#* Known Hosts]	Lifestyle	Notes	
							General	Antibacterial Effects
**Dennis Lab**	KS1 [175]	*Myoviridae*	Unknown (not sequenced)	Onion rhizosphere	*B. cenocepacia* (715J, J2315, K56-2, C6433), *B. ambifaria* (LMG 19467) [7 hosts]	Unknown (not sequenced)		
	DK4/BcepMu/KS4 ^‡^ [175,176,255,263]	*Myoviridae*	*Bcepmuvirus* (*Burkholderia* phage ϕE255)	Lysogen of *B. cenocepacia* J2315	*B. cenocepacia* (K56-2), *B. ambifaria* (LMG 19467) [2 hosts]	Lysogeny-capable	Unstable in lysate, but stable in *G. mellonella* haemolymph [169,175].	
	KS4-M [175]	*Myoviridae*	*Bcepmuvirus* (*Burkholderia* phage ϕE255)	Mutant of DK4/BcepMu/KS4	*B. multivorans* (C5393), *B. cenocepacia* (K56-2, C6433), *B. ambifaria* (LMG 19467) [4 hosts]	Lysogeny-capable	Stable in lysate (unlike parent phage) and in haemolymph. Can be aerosolized and nebulized for inhalatory delivery without significant loss of titer [169,175,264,269,270].	Reduces mortality in *G. mellonella*. Produces a 2.5-log reduction in bacterial density in murine lung infection model [169,170].
	KS5 [175]	*Myoviridae*	*Kisquinquevirus* (*Burkholderia* phage Mana)	Onion rhizosphere	*B. cepacia* (LMG 18821), *B. multivorans* (C5393), *B. cenocepacia* (715J, J2315, K56-2, C6433, C5424), *B. ambifaria* (LMG 19467) [8 hosts]	Lysogeny-capable; integrates at 3′ end of AMP nucleosidase [60].	Primary receptor is LPS (lipid A proximal element is thought to be required) [60].	Decreases bacterial density in murine lung infection model when applied at high MOI, but low MOI has little effect [170].
	KS6 [175]	*Myoviridae*	Unknown (not sequenced)	Onion rhizosphere	*B. cepacia* (LMG 18821), *B. multivorans* (C5393, C5274), *B. cenocepacia* (715J, J2315, K56-2, C6433, C5424), *B. stabilis* (LMG 18870), *B. ambifaria* (LMG 19467) [10 hosts]	Unknown (not sequenced)		
	KS9 [175]	*Siphoviridae*	Unclassified *Siphoviridiae* (*Burkholderia* phage Bcep176)	Lysogen of *B. pyrrocinia* LMG 21824	*B. cenocepacia* (K56-2), *B. ambifaria* (LMG 19467) [2 hosts]	Lysogeny-capable; integrates into 3′ end of GTP cyclohydrase II gene [166].	Primary receptor is LPS (lipid A distal element required). Only Bcc phage that has been converted to obligately lytic form via insertional inactivation of lytic repressor [166].	Reduces mortality in *G. mellonella*; effectiveness of obligately lytic form is not significantly different from that of the wild-type phage [166].
	KS10 [175]	*Myoviridae*	Unclassified *Myoviridae* (*Burkholderia* phage BcepMu)	Lysogen of *B. cenocepacia* K56-2	*B. ambifaria* (LMG 19467), *B. cenocepacia* (PC184), *B. stabilis* (LMG 18870) [3 hosts]	Lysogeny-capable; integrates [260].	Transposable phage [260].	
	KS14 [60]	*Myoviridae*	*Kisquattuordecimvirus* (*Burkholderia* phage FLC5)	*Dracaena* sp. soil	*B. multivorans* (C5393, C5274*), B. cenocepacia* (715J, C6433, C5424, PC184), *B. dolosa* (LMG 21443), *B. ambifaria* (LMG 17828) [8 hosts]	Lysogeny-capable; forms phagemid	Not stable in haemolymph (4-log titer loss in 24 h). Can be aerosolized for use in inhalatory delivery without significant loss of titer [169,270].	Reduces mortality in *G. mellonella*, even at low MOIs. Produces significant reduction in bacterial density in murine lung infection model. Interacts synergistically with ciprofloxacin, meropenem and tetracycline based on plaque diameter [169,170,244].
	KL3 [60]	*Myoviridae*	*Tigrivirus* (*Burkholderia* phage ϕX216)	Lysogen of *B. cenocepacia* CEP511	*B. ambifaria* (LMG 17828) [1 host]	Lysogeny-capable; integrates into threonine tRNA gene		
	DC1 [271]	*Podoviridae*	*Lessievirus* (*Burkholderia* phage BcepIL02*)*	*Dracaena* sp. soil	*B. cepacia* (LMG 18821), *B. cenocepacia* (K56-2, C6433, PC184, CEP511), *B. stabilis* (LMG 18870) [6 hosts]	Lysogeny-capable; integrates; lysogens are unstable	Encodes a CsrA-like protein known to downregulate biofilm formation in *E. coli*. Stable in haemolymph [169,271].	Fails to reduce mortality in *G. mellonella* at any MOI. Reduces bacterial density in murine lung infection model, but is the least efficient Bcc phage tested for this purpose [169,170].
	KL1 [257]	*Siphoviridae*	*Kilunavirus* (*Pseudomonas* phage PaeS_SCUT-S4)	sewage	*B. cenocepacia* (K56-2, C6433, 715J, K63-3) [4 hosts]	Lysogeny-capable	Primary receptor is not LPS. Encodes MazG [257].	
	AH2 [257]	*Siphoviridae*	*Ahduovirus* (*Burkholderia* phage BcepNazgul)	*Nandina* sp. soil	*B. cenocepacia* (K56-2, C6433, 715J, K63-3) [4 hosts]	Lysogeny-capable	Primary receptor is not LPS. Encodes MazG [257].	
	KS12 [169]	*Myoviridae*	Unknown (not sequenced)	*Dietes grandiflora* soil	*B. cenocepacia* (K56-2), *B. multivorans* (C5274) [2 hosts]	Putatively obligately lytic; but not known conclusively due to challenges with sequencing	Not stable in haemolymph (3-log titer loss in 24 h). Likely uses LPS are a primary receptor [169,259].	Reduces mortality in *G. mellonella* and completely clears K56-2 from haemolymph. Persists in murine lungs for at least three days after intraperitoneal or aerosolized delivery. NOID delivery produces significant (2.5-log) reductions in bacterial titer even 3 days after infection. Interacts synergistically with ciprofloxacin, meropenem and tetracycline based on plaque diameter and in vitro liquid growth reduction. Combinations of KS12 with minocycline and meropenem significantly reduce mortality in *G. mellonella* [169,170,244].
	JG068 [259]	*Podoviridae*	*Mguuvirus* (*Ralstonia* phage ϕRSB1)	Sewage	*B. multivorans* (ATCC 17616*), B. cenocepacia* (K56-2, J2315, PC184), *B. stabilis* (LMG 14294), *B. dolosa* (AU0158, CEP021). [6 hosts]	Obligately lytic	LPS inner core is primary receptor [259].	Reduces mortality in *G. mellonella* [259].
	ϕH111-1[261]	*Myoviridae*	Unclassified *Myoviridae* (*Acidiothiobacillus* phage AcaML1)	Lysogen of *B. cenocepacia* (H111)	*B. multivorans* (ATCC 17616, C5274), *B. cenocepacia* (C6433, 715J, K56-2, C1257, C5424, PC184, R161, R452, R750, R1284, R1285, R1314, R1434, R1619, R1882, R1883, R1884, R2314, S11528). [21 hosts]	Lysogeny-capable; integrates into arginine tRNA gene	LPS inner core is primary receptor [261].	
**Young Lab**	Bcep22 [256]	*Podoviridae*	*Lessievirus* (*Burkholderia* phage BcepMigl)	?	*B. cenocepacia* (AU1054) [1 host]	Lysogeny-capable; integrates; lysogens are unstable (likely lost after one generation)		
	BcepIL02 [256]	*Podoviridae*	*Lessievirus* (*Burkholderia* phage DC1)	Corn rhizosphere	*B. cenocepacia* (PC184, AU1054) [2 hosts]	Lysogeny-capable; integrates; lysogens are unstable (likely lost after one generation)		Significantly reduces lung bacterial density in a murine infection model [168].
	BcepB1A [258]	*Myoviridae*	Unclassified *Myoviridae* (*Burkholderia* phage Bupsϕ1	Unspecified soil	*B. cenocepacia* (s198B1A) [1 host]	Obligately lytic		
	Bcep43 [258]	*Myoviridae*	*Naesvirus* (*Burkholderia* phage Bcep781)	Unspecified soil	*B. cenocepacia* (74-34, Bcc43) [2 hosts]	Obligately lytic		
	Bcep1 [258]	*Myoviridae*	*Naesvirus* (*Burkholderia* phage BcepNY3)	Unspecified soil	*B. cenocepacia* (Bcc1) [1 host]	Obligately lytic		
	Bcep781 [258]	*Myoviridae*	*Naesvirus* (*Burkholderia* phage Bcep43)	Unspecified soil	*B. cenocepacia* (74-34, Bcc43) [2 hosts]	Obligately lytic		
**Govan Lab**	NS1 [252]	*Myoviridae*	Unknown (not sequenced)	Lysogen of *B. vietnamiensis* (ATCC 29424)	*B. cepacia* (J2540, C2970), *B. multivorans* (C2775), *B. cenocepacia* (PC184, CEP511, C2836, C3166, LMG 18829, C3170), *B. stabilis* (C3171), *B. vietnamiensis* (ATCC 53266, ATCC 53267, C2973, LMG 16232, C2978), *B. anthina* (J2951, C1765), *B. pyrrocinnia* (C3918, C3930). [19 hosts]	Lysogeny-capable	LPS is likely a primary receptor [252].	
	NS2 [252]	*Myoviridae*	Unknown (not sequenced)	Lysogen of *B. multivorans* ATCC 17616	*B. cepacia* (J2540, C2970), *B. multivorans* (C2775), *B. cenocepacia* (PC184, CEP511, ATCC17765, C1394, J415, BC7, K52-6, C6433, J2315, C2836, C3165, C3166, LMG 18829, C3170), *B. vietnamiensis* (ATCC 53266, ATCC 53267, C2973, LMG 16232, LMG 18836, ATCC 29424). [23 hosts]	Lysogeny-capable	LPS is likely a primary receptor [252].	
	DK1 [255]	*Siphoviridae*	Unknown (not sequenced)	?	*B. multivorans* (C2775), *B. cenocepacia* (J415, LMG 18829, ATCC 17765), *B. vietnamiensis* (C3177), *B. pyrrocinnia* (C3918) [6 hosts]	Lysogeny-capable		
	DK2/DK3 [255]	*Myoviridae*	Unknown (not sequenced)	Lysogen of *B. cenocepacia* (C3166) and *B. stabilis* (C3174)	*B. cepacia* (ATCC 17759), *B. multivorans* (C2775), *B. cenocepacia* (C1394, J2956, LMG 18829), *B. stabilis* (C3173), *B. vietnamiensis* (C3177) [7 hosts]	Lysogeny-capable		
	JB1 [255]	*Myoviridae*	Unknown (not sequenced)	Unspecified soil	*B. cepacia* (C2970), *B. cenocepacia* (J2956, LMG 18829, ATCC 17765), *B. stabilis* (LMG 18870), *B. vietnamiensis* (C3177), *B. anthina* (J2951, C1658, C1765), *B. pyrrocinnia* (C3909, C3918, C3930, C3993, C3995, C3997), *B. ubonensis* (E551). [16 hosts]	Unknown (not sequenced)		
	JB3 [255]	*Siphoviridae*	Unknown (not sequenced)	Unspecified plant rhizosphere	*B. multivorans* (C2775), *B. cenocepacia* (J2956, LMG 18829), *B. anthina* (C1658, C1765), *B. ubonensis* (E551) [6 hosts]	Unknown (not sequenced)		
	JB5 [255]	*Myoviridae*	Unknown (not sequenced)	Unspecified plant rhizosphere	*B. cepacia* (C2970), *B. cenocepacia* (J2956, C2836, LMG 18829, ATCC 17765), *B. vietnamiensis* (C3175, C3177), *B. anthina* (J2951, C1658, C1765), *B. pyrrocinnia* (C3909, C3918, C3830, C3993, C3995, C3997). [16 hosts]	Unknown (not sequenced)		
	RL1c [255]	*Myoviridae*	Unknown (not sequenced)	Unspecified plant rhizosphere	*B. cepacia* (C2970), *B. multivorans* (C2775), *B. cenocepacia* (J2956, ATCC 17765), *B. stabilis* (C3171), *B. vietnamiensis* (C2978, C3177), *B. anthina* (C1765). [8 hosts]	Unknown (not sequenced)		
	RL2 [255]	*Myoviridae*	Unknown (not sequenced)	Pond sediment	*B. cenocepacia* (J415, C1394, J2956, C2836, C3169, C3170), *B. vietnamiensis* (C3177), *B. anthina* (J2951, J2862*), B. pyrrocinnia* (C3918, C3930, C3993, C3995), *B. ubonensis* (E26). [15 hosts]	Unknown (not sequenced)		
**Others**	CP75 [272]	*Myoviridae*	Unknown (not sequenced)	Lysogen of *Pseudomonas cepacia* (PCT1)	No known hosts	Lysogeny-capable	First identified phage of the Bcc [272].	
	BcP15 [273,274]	*Siphoviridae*	Unknown (not sequenced)	Lysogen of *B. cepacia* DR11	No known hosts	Lysogeny-capable		
	AP3 [265]	*Myoviridae*	*Kisquinquevirus* (*Burkholderia* phage Mana)	Water sample	*B. cenocepacia* (5, 6, 10, 18, 20, 21, 7780, 1567, 1947, 39) [10 hosts]	Lysogeny-capable; integrates into tRNA genes		Reduces mortality in *G. mellonella*. [265]
	G4P1 [275]	*Myoviridae*	Unclassified *Myoviridae* (*Manheimia haemolytica* phage 3927AP2)	Lysogen of *B. vietnamiensis* G4	*B. ambifaria* (J82, R-8863, ATCC 51671, LMG-P 24640, LMG 17828, MVP/C1 64, MC40-6), *B. cenocepacia* (LMG 18829), *B. contaminans* (CEP0964), *B. dolosa* (AU0794, AU3556, AU1568, AU3960, AU4298, AU2130), *B. vietnamiensis* (LMG 18835, LMG 10929; CEP1224). [18 hosts]	Lysogeny-capable; integrates into arginine tRNA gene		

* Bcc phages that do not appear in any publication, or those for that only the genome has been published, do not appear in this table. † Citations listed in this column are the main source of information on the given phage, unless other citations are provided in specific columns. ‡ This phage was first isolated as DK4 (Govan lab), then as BcepMu (Young lab), and finally as KS4 (Dennis lab). It appears in this section to show contrast between it and its mutant, KS4-M, which was isolated only by the Dennis lab and appears in the next row.

Furthermore, bacterial surface structures, such as flagella, pili, and adhesins, assist the Bcc bacteria with motility and adherence to host cells, and are essential for invasion of the epithelial cells of the respiratory tract [229]. Aside from their primary role in bacterial motility, flagella are known to be involved in bacterial pathogenesis, and flagellin-deficient mutants of the *B. cenocepacia* type strains J2315 and K56-2 have repeatedly been found to be avirulent, and had significantly reduced invasion capabilities. In a murine model of infection, the flagellin-mutated K56-2 strain was harmless and produced no fatalities, while 40% of the mice infected with the wild-type K56-2 strain died by three days post-infection [229,276]. Five types of pili have thus far been identified in the Bcc species, but only cable pili have been associated with endemic strains of *B. cenocepacia*. These cable pili can tether aggregates of cells, increase adherence to epithelial cells, and, together with adhesins such as AdhA, bind intermediate filament proteins such as cytokeratin-13 (CK13), which are used as receptors for entry into host cells, thus assisting *B. cenocepacia* strains to establish infections [277]. Although no Bcc phages that are known to attach to these structures have been identified as of yet, these surface structures frequently serve as primary attachment sites for phages infecting other species, and are likely bound by some Bcc phages as well [158,173]. The use of such phages as a selection pressure could push targeted Bcc populations towards less-virulent phenotypes, thus reducing the mortality associated with Bcc infections.

Synergistic interactions between antibiotics and Bcc-targeting bacteriophages have been described in only one report, in which the *myoviridae* KS12 and KS14 were shown to interact synergistically with several antibiotics that are commonly used against the Bcc, including meropenem, tetracycline, and ciprofloxacin [244]. Indeed, at both inhibitory and subinhibitory concentrations of these compounds, the plaques of KS12 and KS14 became significantly larger than when no antibiotics were present. Furthermore, combinations of KS12 with subinhibitory concentrations of these three antimicrobials were significantly more effective than individual treatments with either the phage or any of the antibiotics, producing nearly four-log reductions in the growth of the *B. cenocepacia* strain K56-2, relative to untreated controls, in liquid culture. Finally, combined treatment, using KS12 and either meropenem or minocycline, significantly reduced mortality in *B. cenocepacia* K56-2-infected waxworms, more so than treatment with either of the two agents individually, further highlighting the efficacy of this combined form of treatment. Interestingly, the treatment of log-phase cells with ciprofloxacin, meropenem, and tetracycline was found to produce cell filamentation, elongation, and clustering, respectively; the former two might account for the observed synergistic effects—since increased cell surface area could lead to increased burst sizes via the ‘Delayed Lysis’ mechanism [244,250]. As KS14 is temperate, its synergistic interaction with ciprofloxacin could also be explained by the ‘Induction’ mechanism, since ciprofloxacin is known to activate the bacterial stress response and could therefore revert KS14 prophages to the lytic cycle [164,244].

Although no studies seeking to exploit the phage–antibiotic catch-22 mechanism, described by Chan et al., have been conducted with Bcc phages as of yet, this might be another promising phage–antibiotic strategy against the Bcc. Since efflux pumps such as those of the RND superfamily play a major role in the antimicrobial resistance of the Bcc species, combinations of efflux pump-binding phages and commonly extruded antibiotics would be therapeutically potent against many of these species [105,209]. No Bcc phages that are known to utilize efflux pumps as their primary receptors have been reported, however, underscoring the importance of continued bacteriophage isolation and characterization. 

Finally, a promising target of phage–antibiotic cocktails against the Bcc species is the Bcc biofilm, which plays major roles in the virulence, immune evasion, and antimicrobial resistance of many Bcc species. Indeed, when comparing the susceptibility of planktonic- and biofilm-grown Bcc strains to various antibiotics, Desai et al. observed a 15-fold increase in the resistance to ciprofloxacin and ceftazidime among biofilm-grown cells relative to their planktonic counterparts, demonstrating that Bcc-secreted exopolysaccharides (EPS), critical components of biofilms, are capable of forming protective barriers that confer resistance to antibiotics [278]. The Bcc species are known to produce at least the following four types of EPS: PS-I, cepacian (PS-II), levan, and an as-of-yet unnamed biopolymer, with distinct strains producing different amounts of each of these compounds [279,280]. The vast majority of Bcc strains—including both clinical and environmental isolates—have been found to produce cepacian, a branched heptasaccharide that is known to increase virulence [281]. Several studies have recently demonstrated that *B. cenocepacia* mutants producing increased amounts of cepacian are virulent and have increased persistence in murine infection models, which is largely due to the fact that cepacian interferes with neutrophil-mediated phagocytosis [282,283]. Additionally, cepacian was recently found to inhibit the production of chemoattractants and ROS in neutrophils, which consequently inhibits the recruitment of immune cells and neutrophil-mediated killing of bacteria, meaning that cepacian–and EPS in general–are heavily implicated in the exacerbation of Bcc infections [57,283]. Although antibiotic penetration into biofilms is generally poor, many phages encode exopolysaccharide-degrading depolymerases, which are able to degrade the outer layers of biofilms, and thus permit the subsequent killing (by phage and accompanying antibiotics) of the bacteria within. Such phages could be used to eradicate biofilms and possibly provide a selection pressure to downregulate the production of immunosuppressing EPS such as cepacian. Although no studies investigating the effectiveness of Bcc phages in reducing Bcc biofilms, either alone or in combination with relevant antibiotics, have been conducted as of yet, the Bcc *podovirus* DC1 is known to encode a polypeptide homologous to CsrA, a regulator protein known to repress biofilm formation in *E. coli*, meaning that this phage could potentially be combined with antibiotics into cocktails designed specifically to infiltrate and degrade Bcc biofilms [212,271].

### 4.3. Experimental Bcc Phage Therapy In Vivo

Although human clinical trials have yet to be conducted for Bcc phage therapy, several promising studies investigating the ability of bacteriophages to treat Bcc infections in vivo have been conducted, with two different model organisms. The simplest and most commonly utilized in vivo model for Bcc phage therapy is the *Galleria mellonella* (greater waxworm) larva model, in which bacteria and phages are sequentially injected into larvae and the treatment efficacy is determined by the survival of infected larvae up to 48 h post-infection. Although *G. mellonella* lack adaptive immune systems and have relatively rudimentary internal compartmentalization–and are therefore imperfect analogues of higher mammals—they nevertheless serve as an excellent preliminary model through which to evaluate the usefulness of specific phages in vivo [284]. The model was initially tested through the administration of lethal doses of the *B. cenocepacia* strains C6433 and K56-2, and increased larval survival and rescue from bacterial infection was observed for larvae treated through monophage therapy with the *myoviridae* KS4-M, KS12, and KS14, as well as the *podovirus* JG068 and the *siphovirus* KS9. Due to its simplicity, the waxworm model has become popular for preliminary testing of Bcc phage therapy, and was recently used to demonstrate the in vivo effectiveness of the *B. cenocepacia*-specific *myovirus* AP3 [166,169,259,265]. Interestingly, the genetically engineered constitutively lytic variant of the normally lysogeny-capable KS9, dubbed KS9c, did not exhibit an improved ability to rescue *B. cenocepacia*-infected *G. mellonella*, possibly suggesting that resistance through superinfection immunity plays a lesser role in in vivo phage–bacteria dynamics than had been previously anticipated [166]. Despite their therapeutic effectiveness, KS12 and KS14 appear to be unstable in *G. mellonella* hemolymph, experiencing 3–4-log reductions in titer over 24 h, while the *podovirus* DC1 exhibits hemolymph stability, but produces no reductions in mortality–possibly suggesting that in vivo stability and therapeutic effectiveness may be negatively correlated in some phages [169]. Although a precise mechanistic understanding of this phenomenon remains elusive, one possibility is that KS12 and KS14 are highly immunostimulatory and therefore recruit innate immune cells that remove them from circulation, but also eliminate infecting bacteria (see Section 3.4), thereby accounting for both therapeutic effectiveness and decreased titer in the hemolymph. *G. mellonella* is the only organism in which Bcc phage–antibiotic synergy has been tested, and it was demonstrated that although KS12, minocycline and meropenem produce appreciable reductions in mortality when used individually, a combination of KS12 with either of these two antibiotics is superior and significantly improves survival in *B. cenocepacia*-infected larvae [244].

A more complicated model of Bcc phage therapy is the murine lung infection model, in which bacteria and phages are sequentially administered to the lungs of mice, and the titers of both bacteria and phages are subsequently measured to determine the efficacy of the treatment [168,170]. In a study by Carmody et al., mice were infected with *B. cenocepacia* via tracheotomy, and the *podovirus* BcepIL02 was administered 24 h post-infection, by either intranasal inhalation or intraperitoneal injection. Mouse lungs were assayed for bacteria and phage titers 48 h post-infection, and a significant reduction in Bcc titers was observed for all the treated mice, although the reductions were significantly larger in the mice treated intraperitoneally relative to those receiving treatment via inhalation. In contrast, Semler et al. demonstrated that phage delivery via aerosol inhalation produces greater decreases in bacterial titer relative to intraperitoneal treatment. In this study, chemically immunocompromised mice that were infected with *B. cenocepacia* were treated through monophage therapy with one of KS12, KS14, KS5, KS4-M, and DC1, and both bacterial and phage titers were monitored two and three days post-infection. Significant, four-fold decreases in bacterial titers were observed for the mice treated via aerosol inhalation relative to the mice receiving intraperitoneal treatments, as well as the infected, saline-treated controls, and the bacterial titers of aerosol phage-treated mice remained low, even after four days post-infection. Importantly, both studies demonstrated that UV- or heat-inactivated phages produced no reductions in bacterial titer, indicating that the therapeutic effectiveness of these phages is due to the killing of bacterial cells rather than the direct activation of the host immune system, regardless of the immunocompetence status of the host organism. Moreover, Carmody et al. demonstrated that neither inhalatory nor intraperitoneal administration of BcepIL02 resulted in increased production of TNF-α or the neutrophil chemokine MIP-2, suggesting that at least some Bcc phages do not trigger induction of the inflammatory response. Although a mechanistic understanding of the differences in effectiveness of the two treatments utilized in these studies remains elusive, Carmody et al. suggests that one reason why the inhalatory delivery of BcepIL02 is less effective in their study is that this phage is unable to efficaciously transcytose directly into the lung parenchyma, where the majority of bacterial killing is expected to take place. Since different phages may move through the body tissues at different rates, the problem encountered by the inhaled delivery of BcepIL02 may not occur for some other phages, possibly accounting for the superior efficacy of inhaled phage treatment in the report by Semler et al. Alternatively, the effectiveness of different modes of treatment may depend on the portion of the lungs that is colonized by bacteria, as some lung compartments may be differentially accessible to inhaled and intraperitoneally injected phages. In both cases, however, bacterial burden was greatly reduced by the administration of phages, demonstrating the in vivo effectiveness of Bcc phage therapy [168,170,205].

As of 2021, only one case report involving the compassionate care phage treatment of a patient afflicted with a multidrug-resistant Bcc strain has been published [135]. In this case, a CF patient infected with an MDR strain of *B. dolosa*, sensitive only to minocycline, received a bilateral lung transplant. The patient’s bilateral pleural spaces and endobronchial cultures remained infected with *B. dolosa* after the operation, despite extensive multidrug therapy, and recurrent pneumonia developed approximately five months following transplant. At six months post-transplant, a single lytic phage was combined with the drug regimen, and the patient rapidly exhibited decreased fever, leukocytosis, and airway secretions, and the *B. dolosa* burden was greatly reduced upon culture of bronchoalveolar lavage (BAL). Approximately nine months post-transplant, however, progressive renal and hepatic failure, due to antibiotic toxicity, led to a cessation of treatment, which resulted in increased BAL growth, resurgence of pneumonia, and eventual death 11 months following the transplant. In this case, it appears that although phage therapy improved the patient’s condition and overall clinical outlook, toxicity caused by the long-term use of antibiotics led to drug-induced injury and subsequent fatality [135]. Although this patient succumbed, this case highlights the necessity of utilizing phages in concert with antibiotics from the onset of treatment, rather than as a last resort, since such a combined treatment may reduce the long-term dependence on antibiotics, and thereby mitigate the toxicity associated with their extensive use. 

## 5. Summary and Future Directions

The species that comprise the *B. cepacia* complex can cause chronic and often life-threatening lung infections in susceptible populations, notably patients afflicted with CF and CGD, and are notoriously challenging to eradicate, due to a high degree of intrinsic antimicrobial resistance—thereby necessitating the development of a novel mode of treatment. Although phage therapy has a checkered history and has frequently been met with academic skepticism, which was perhaps appropriate in the early years of its renaissance, an enormous volume of modern research has been amassed to demonstrate that phage therapy is efficacious, safe, environmentally friendly, cost effective, versatile, and, in many aspects, outright superior to antibiotic therapy. Moreover, the concomitant use of phages and antibiotics can improve the efficacy of both of these components, while simultaneously relieving at least some of the toxicity associated with the excessive use of chemical antimicrobials. Crucially, the use of specific, scientifically supported treatment strategies may lead to decreased phage resistance, re-sensitization to chemical antimicrobials, reduction in virulence, and thus high mortality that is associated with many bacterial infections. Among the Bcc, phage therapy has been shown to be highly effective in several animal models, and preliminary investigations have validated phage–antibiotic combinations as capable of efficaciously eradicating these problematic species.

In the next decade, Bcc phage therapy research ought to focus on increasing understanding of Bcc phage receptors, with the aim of identifying efflux pump and virulence factor-targeting phages, which could be used in specific therapeutic strategies, as well as elucidating the mechanisms of phage–antibiotic and potentially phage–phage, synergy with which to optimize the construction of efficacious polyphage–antibiotic cocktails. Importantly, relatively little is known about the interaction of Bcc phages with the mammalian immune system—including the immunostimulatory potential of Bcc phages and whether this helps or hinders therapy—which is an area that requires further exploration if Bcc phages are ever to be used therapeutically. These investigations must be increasingly accompanied by murine and other mammalian trials, which validate the ability of polyphage–antibiotic medicines to resolve Bcc infections in vivo. Finally, governing bodies must modify the existing legislation to allow for improved, timely, holistic, and scientifically informed approaches to employing experimental phage therapy in Bcc-afflicted patients who need it, in order to reduce mortality and improve overall quality of life.

## Data Availability

Not applicable.
